# Monoacylglycerol lipase regulates macrophage polarization and cancer progression in uveal melanoma and pan-cancer

**DOI:** 10.3389/fimmu.2023.1161960

**Published:** 2023-03-23

**Authors:** Yao Tan, Juan Pan, Zhenjun Deng, Tao Chen, Jinquan Xia, Ziling Liu, Chang Zou, Bo Qin

**Affiliations:** ^1^ Shenzhen Aier Eye Hospital, Aier Eye Hospital, Jinan University, Shenzhen, China; ^2^ National Center for International Research of Bio-targeting Theranostics, Guangxi Key Laboratory of Bio-targeting Theranostics, Collaborative Innovation Center for Targeting Tumor Diagnosis and Therapy, Guangxi Talent Highland of Bio-targeting Theranostics, Guangxi Medical University, Nanning, Guangxi, China; ^3^ Department of Clinical Medical Research Center, The Second Clinical Medical College, The First Affiliated Hospital of Southern University of Science and Technology, Jinan University (Shenzhen People’s Hospital), Shenzhen, Guangdong, China; ^4^ Department of Dermatology, The Second Clinical Medical College, Jinan University (Shenzhen People’s Hospital), Shenzhen, China; ^5^ The First Affiliated Hospital, Jinan University, Guangzhou, China; ^6^ School of Medicine, The First Affiliated Hospital, Southern University of Science and Technology, Shenzhen, China; ^7^ Institute of Biopharmaceutical and Health Engineering, Tsinghua Shenzhen International Graduate School, Tsinghua University, Shenzhen, China; ^8^ School of Life and Health Sciences, The Chinese University of Kong Hong, Shenzhen, China; ^9^ Shenzhen Aier Ophthalmic Technology Institute, Shenzhen, China

**Keywords:** uveal melanoma (UM), lipid metabolism, cancer prognosis, tumor microenvironment (TME), macrophage polarization, monoacylglycerol lipase (MGLL)

## Abstract

**Background:**

Although lipid metabolism has been proven to play a key role in the development of cancer, its significance in uveal melanoma (UM) has not yet been elucidated in the available literature.

**Methods:**

To identify the expression patterns of lipid metabolism in 80 UM patients from the TCGA database, 47 genes involved in lipid metabolism were analyzed. Consensus clustering revealed two distinct molecular groups. ESTIMATE, TIMER, and ssGSEA analyses were done to identify the differences between the two subgroups in tumor microenvironment (TME) and immune state. Using Cox regression and Lasso regression analysis, a risk model based on differentially expressed genes (DEGs) was developed. To validate the expression of monoacylglycerol lipase (MGLL) and immune infiltration in diverse malignancies, a pan-cancer cohort from the UCSC database was utilized. Next, a single-cell sequencing analysis on UM patients from the GEO data was used to characterize the lipid metabolism in TME and the role of MGLL in UM. Finally, *in vitro* investigations were utilized to study the involvement of MGLL in UM.

**Results:**

Two molecular subgroups of UM patients have considerably varied survival rates. The majority of DEGs between the two subgroups were associated with immune-related pathways. Low immune scores, high tumor purity, a low number of immune infiltrating cells, and a comparatively low immunological state were associated with a more favorable prognosis. An examination of GO and KEGG data demonstrated that the risk model based on genes involved with lipid metabolism can accurately predict survival in patients with UM. It has been demonstrated that MGLL, a crucial gene in this paradigm, promotes the proliferation, invasion, and migration of UM cells. In addition, we discovered that MGLL is strongly expressed in macrophages, specifically M2 macrophages, which may play a function in the M2 polarization of macrophages and M2 macrophage activation in cancer cells.

**Conclusion:**

This study demonstrates that the risk model based on lipid metabolism may be useful for predicting the prognosis of patients with UM. By promoting macrophage M2 polarization, MGLL contributes to the evolution of malignancy in UM, suggesting that it may be a therapeutic target for UM.

## 1 Introduction

Uveal melanoma (UM) is the most prevalent primary intraocular malignant tumor and the second most prevalent kind of malignant melanoma (1), originating in the iris, choroid, and ciliary body (2, 3). Although UM and cutaneous melanoma are melanocyte-derived malignant tumors, UM has distinct clinical and biological characteristics (4). UM, a rare malignancy is most common in non-Hispanic whites with lighter skin and blue eyes (5). A recent meta-analysis revealed that the incidence rates in North America, Europe, and Asia were 5.74 (95% CI: 4.37-7.11), 7.3 (95% CI: 6.36-8.24), and 0.53 (95% CI: 0.31-0.74) respectively (6). The onset of UM is associated with some risk factors including fair skin color, light eye color, ability to tan, oculodermal melanocytosis, nevi, and BRCA1-associated protein 1 (BAP1) mutation (7). Nowadays, enucleation and radiotherapy—plaque and proton beam—are the most widely used treatments for UM (8, 9). A gene expression profile study divided UM patients into two kinds (low metastatic risk and high metastatic risk) (10). Only 15% of advanced (metastatic) UM patients have a one-year survival rate, and median survival varies from 4 to 15 months (11). A psychological test found that nearly all UM patients desire to know their survival prognosis at the time the tumor was diagnosed (12). An increasing number of research have been focused to elucidate the genetic and pathological mechanisms involved in UM prognosis, however precise prognostication for patients is far from unattainable (13, 14). Currently, there is a huge need to investigate important indicators that can provide reassurance to patients with a high chance of better survival or provide counseling, screening, and systemic adjuvant therapy to patients at high risk (15–17). Consequently, it is imperative to identify a risk classification strategy and prognostic genes for the development of personalized therapy for UM patients.

Due to the rapid proliferation of tumor cells and inadequate blood vessels formation, the tumor microenvironment (TME) is characterized by hypoxia, high oxidation, acidity, and malnutrition, therefore tumor cells reshape their microenvironment *via* multiple processes including lipid metabolic reprogramming (18, 19) to sustain unrestricted cell proliferation and survival. Metabolic reprogramming has been considered a hallmark of cancer for its ability to adapt TME, and dysregulation of lipid metabolism has been a focal point of recent research (20), contributing to the progression of various cancers including glioblastomas, prostate cancer, breast cancer, hepatocellular carcinoma, pancreatic cancer (21–25). Numerous studies have demonstrated that alterations in tumor lipid metabolism led to tumor formation and immunosuppression in the TME (26). Increasing evidence suggests a significant role of lipid metabolism in melanoma pathogenesis (27). However, the function of lipid metabolism-related genes (LMRGs) in determining the outcome of UM is not well understood.

In this study, we sought to identify key LMRGs associated with TME in UM and to construct a predictive model for UM. This project seeks to find novel prognostic indicators and therapeutic targets as well as clarify the condition of the tumor immune microenvironment in UM in order to build a molecularly-based technique for predicting survival and treatment advantages for UM patients.

## 2 Methods

### 2.1 Data collection

The Cancer Genome Atlas (TCGA) provided UM patients’ clinicopathological characteristics and gene expression matrices. The training cohort included 80 UM patients (28). The validation cohort (containing 28 cases from GSE84976) was derived from the Omnibus (GEO) datasets (29). Clinical information included survival time, survival status, gender, age, tumor grade, and stage. Missing clinical information samples were eliminated.

LMRGs were chosen from Gene Set Enrichment Analysis (GSEA) and Kyoto Encyclopedia of Genes and Genomes (KEGG) databases. GSEA has 17 lipid metabolism-related gene sets, while KEGG has 16. **Table S1** lists GSEA and KEGG gene sets. 1168 LMRGs remained for research after eliminating duplication genes.

### 2.2 Consensus clustering

The R package “ConsensusClusterPlus” (version 1.54.0) was used to classify UM patients into two subgroups based on the 47 LMAG expression matrix (30, 31). LMRGs consensus clustering analysis found the optimal number of clusters, the lowest fraction of ambiguous clusterings, and the best CDF value for k = 2.

### 2.3 Calculation of microenvironment cell abundance

The “ESTIMATE” R package (version 1.0.13) calculated ESTIMATE scores, immune scores, stromal scores, and tumor purity (32). microenvironment cell populations (MCPs) and immune cells were quantified using transcriptomic data from the “MCPcounter” package in R (version 1.1) (33). Single sample gene set enrichment analysis (ssGSEA) was performed using the R package “GSVA” (version 1.24.0) to evaluate 28 immune infiltrating cell types (34, 35).

### 2.4 Differential gene expression and functional enrichment analysis

“limma” R package (version 3.40.6) performed differential gene expression analysis. We selected differentially expressed genes (DEGs) using |log2(fold change)| >1 and a false discovery rate (FDR) adjusted p < 0.05. The DEGs list was then analyzed using Gene Ontology (GO) and KEGG *via* “clusterProfiler” R package (version 4.4.4). Enrichment analysis and protein-protein interaction (PPI) analyses were done with “Metascape” (36). Using the molecular signature database’s “GO biological process” gene set, the “GSVA” R program (version 1.24.0) investigated the two clusters’ signaling pathways. GSEA analyzed the significant pathways.

### 2.5 Construction of the immune-related risk model

The R package “glmnet” (version 4.1-2) and absolute shrinkage and selection operator algorithm (LASSO) analysis generated a risk model based on univariable regression analysis of prognostic genes. The smallest lambda value was ideal. Multivariate Cox regression analysis determined the gene risk model. The following formula calculated the risk score: risk score = - 0.154969330859525 * expression value of ectonucleotide pyrophosphatase 2 (ENPP2) + 0.168756185717411 * expression value of MGLL - 0.491974590575217 * expression value of phospholipase C delta 1 (PLCD1) - 0.329592494818697 * expression value of solute carrier family 44 member 3 (SLC44A3). The lipid‐related gene signature risk score evenly divided patients into low‐risk and high‐risk groups.

**Figure 1:**
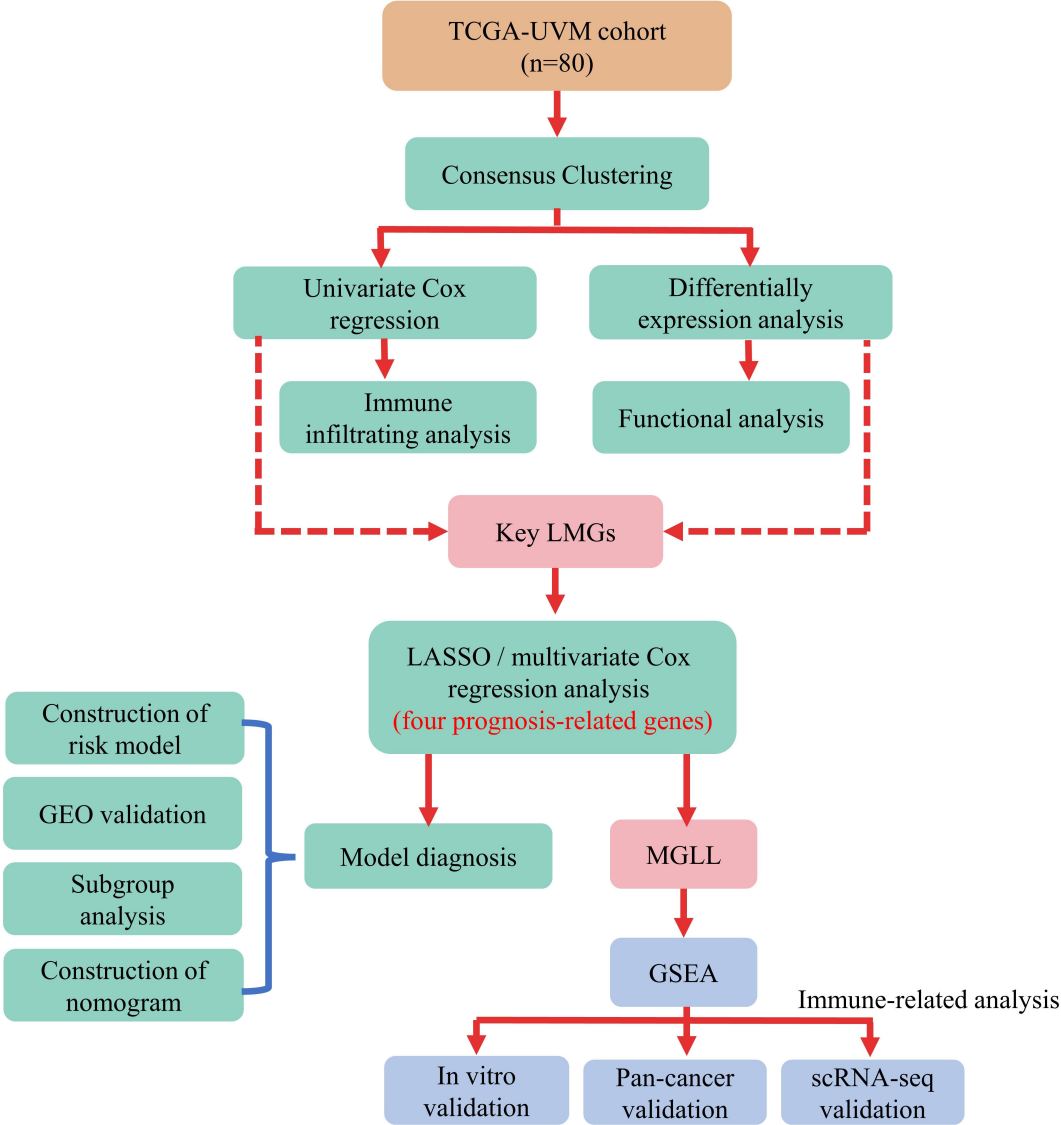
Flow chart of the data analysis procedure.

Kaplan-Meier analysis in R package “survival” (version 3.2-7) assessed the survival difference between two subgroups. Time-dependent receiver operating characteristic (ROC) curve analysis using the “survivalROC” R package (version 1.0.3) was also used to verify the risk model’s prediction accuracy. **Figure 1** illustrates the data analysis procedure.

### 2.6 Pan-cancer analysis of MGLL expression

The pan-cancer data set was obtained from the UCSC database and retrieved monoacylglycerol lipase (MGLL) gene expression data from each sample. Using the TIMER2 database (http://timer.cistrome.org/), pan-cancer macrophage infiltration and MGLL mRNA expression were correlated (37). Reassessed pan-cancer patient immune cell infiltration score *via* the R package “IOBR” (version 0.99.9) QUANTISEQ (38). Survival data were integrated by sample barcode to examine MGLL mRNA expression in pan-cancers. The median value of MGLL expression was utilized to distinguish high- and low-expressing tumor samples. Using the R package “survival” (version 3.2-7), survival time and status were fitted within the two groups. Cox proportional hazards models and log-rank tests examined the correlation between MGLL mRNA expression and overall survival (OS), disease-specific survival (DSS), disease-free survival (DFS), and progression-free survival (PFS). All cancer types are abbreviated in **Table S2**.

### 2.7 Single-cell RNA-seq online analysis

The scTIME Portal (http://sctime.sklehabc.com/unicellular/home) is a database with single-cell time-specific analytic tools for exploring and analyzing TIMEs (39). The “NormalizeData” function, the LogNormalize procedure, and a scale factor of 10,000 were used to normalize the GSE139829 dataset before nonlinear dimension reduction with the “RunUMAP” function and a dims parameter of “1:30”. UM patients’ cell type proportions and connections were then determined.

### 2.8 Cell lines and cultures

The human UM cell line MuM-2B (iCELL-h148; Shanghai, China) (40) and the human monocytic leukemia THP-1 cells (#TIB202; ATCC, USA) were grown in RPMI-1640 media containing 10% FBS and 1% penicillin-streptomycin (Gibco, USA) at 37°C and 5% CO2. The adult retinal pigment epithelial cell line (ARPE-19) cells from American Type Culture Collection (ATCC, Manassas, VA, USA) were cultured in DMEM/F12 (Gibco, USA). Shanghai Baoyi Applied Biotechnology Co., Ltd. did a short tandem repeat (STR) analysis. Experiments were done during logarithmic cell growth.

### 2.9 Monoacylglycerol lipase small interfering RNA construction and transfection

MGLL-knockdown small interfering RNA (siRNA) and negative control siRNA were purchased from Guangzhou RiboBio Co., Ltd. MGLL siRNA was transfected into MuM-2B cells by Lipofectamine RNAi Max (Invitrogen, CA, USA) to transient knockdown MGLL. Western blotting confirmed siRNA inhibition after 48 h of transfection. The siRNA sequence is CCAGGACAAGACTCTCAAGAT (41).

### 2.10 Cell proliferation assay

Cell proliferation experiment was performed using Cell Counting Kit-8 (CCK-8) (MedChem Express, Monmouth Junction, NJ, USA). 2000 MuM-2B cells per well were seeded into 96-well plates transfected with MGLL or negative control siRNA. After seeding for 24 h, 48 h, 72 h, 96 h, and 120 h, each well received 10 μL CCK-8 solution and was incubated at 37 °C for 1.5 h in the dark. A microtiter plate reader (BIO-TEK Instruments, Winooski, VT, USA) measured live cells at 450 nm.

### 2.11 Migration and invasion assays

The cell invasion assay used Matrigel (BD Biosciences, Mississauga, Canada), while the cell migration assay did not. 4 × 10^4 cells per well suspended in 200 μL serum-free medium were added to the cell culture insert (24-well insert, 8-μm pore size), and 500 μL 10% FBS-supplemented RPMI-1640 were added to the well to stimulate cell migration or invasion. After 24 hours, the cells in the inserts were removed and the cells that penetrated and attached to the bottom membrane were fixed with 4% paraformaldehyde (PFA) and stained with crystal violet (0.05% [w/v]). A photomicroscope took images in three randomly selected fields in each well.

### 2.12 Scratch wound healing assay

MuM-2B cells were seeded at 5 × 10^5 cells per well in a 6-well microplate. Scratches were made in the middle of the well with a sterile 200 μL pipette tip when cells reached 95% confluence. Serum-free medium replaced 10% FBS-supplemented RPMI-1640. Photographs were taken at 0 h, 24 h, and 48 h to estimate gap closing. 2.13 RNA extraction and real-time quantitative PCR (RT-qPCR) assays

TRIzol Reagent (Invitrogen, USA) extracted total RNA from cells, and cDNA was generated from 1 μg of RNA using the M-MLV Reverse Transcriptase Kit (Promega, USA) according to the manufacturer’s instructions. A Bio-Rad iQ5 RT-qPCR System performed RT-qPCR. GAPDH normalized transcript expression. **Table S3** lists the primer sequences used in this study.

### 2.14 Western blot

The total cell protein was isolated using radio immunoprecipitation assay buffer (RIPA; Beyotime, China) and quantified using BCA Protein Assay Kits (Pierce, Rockford, IL, USA). SDS-PAGE separated the identical protein samples, which were electro-transferred into PVDF membranes (Millipore Corp, Atlanta, GA, USA). After 1 hour of blocking in 5% non-fat milk, anti-MGLL (1:1000 dilution, #A6654, ABclonal, China) and anti-β-actin (#4970, Cell Signaling Technology, USA) primary antibodies were incubated overnight at 4°C. HRP-conjugated secondary antibodies (Cell Signaling Technology, USA) were incubated for 1 h at room temperature. Thermo Fisher ECL reagents detected band signals. It is recommended that strips be washed with stripping buffer (P0025, Beyotime, China) to ensure that the previous antibody has been removed and imaging can be repeated if necessary.

### 2.15 THP-1 polarization

THP-1 cells were seeded at 1 x 10^6 per well in 6-well plates and treated with PMA (100 nmol; Sigma-Aldrich, St. Louis, MO, USA) for 48 h. M1 macrophages were polarized by incubation with IFN-gamma (20 ng/mL; R&D System, USA) and LPS (100 ng/mL; Sigma, USA) for 48 h. IL-4 (100 ng/ml; PeproTech) was added for 48 h to elicit M2-phenotype polarization.

### 2.16 Statistical analysis

The data were analyzed using R (version 4.0.3) and GraphPad Prism (version 8.0.1). Univariate and multivariate Cox proportional hazards regression identified independent prognostic factors. After multivariate Cox regression analysis, a nomogram was created to predict 3-year RFS and validated with C‐index. Following the regrouping of patients by age, sex, and metastasis, a subgroup analysis was conducted. Student’s t-test was used for statistical analysis between two groups, however one-way ANOVA was applied when there were three or more groups. *P* < 0.05 was statistically significant.

## 3 Results

### 3.1 Two LMRGs-based molecular subtypes and their prognostic significance

**Figure 2:**
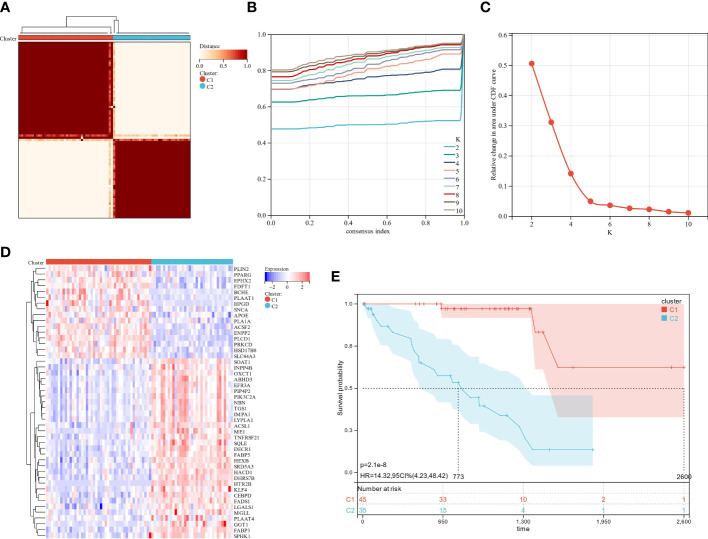
The analysis of consensus clustering. **(A–C)** Consensus clustering is best performed with K = 2. **(D)** A heatmap illustrating the differential expression of lipid metabolism genes between the two groups. **(E)** Two subgroups of patients displayed different survival curves.

Gene expression profiles and clinical data for 80 UM patients were gathered from the TCGA data portal. First, univariate Cox analysis screened gene sets linked with UM patient survival, then 47 LMRG-associated genes were selected (**Table S4**). Unsupervised consensus clustering established the optimal number of groups (k = 2) based on the expression patterns of 47 survival-related LMRGs genes (**Figures 2A–C**). We found two kinds of UM patients: cluster 1 (45, 56.25%) and cluster 2 (35, 43.75%). These survival-related LMRGs genes in the two clusters differed, as shown by the heatmap (**Figure 2D**). Cluster 2 had a substantially lower OS rate than cluster 1 (**Figure 2E**, P < 0.001). These data showed that LMRGs are greatly linked to UM patients’ overall survival.

### 3.2 Identification of differentially expressed lipid metabolism-related genes and functional annotation

**Figure 3:**
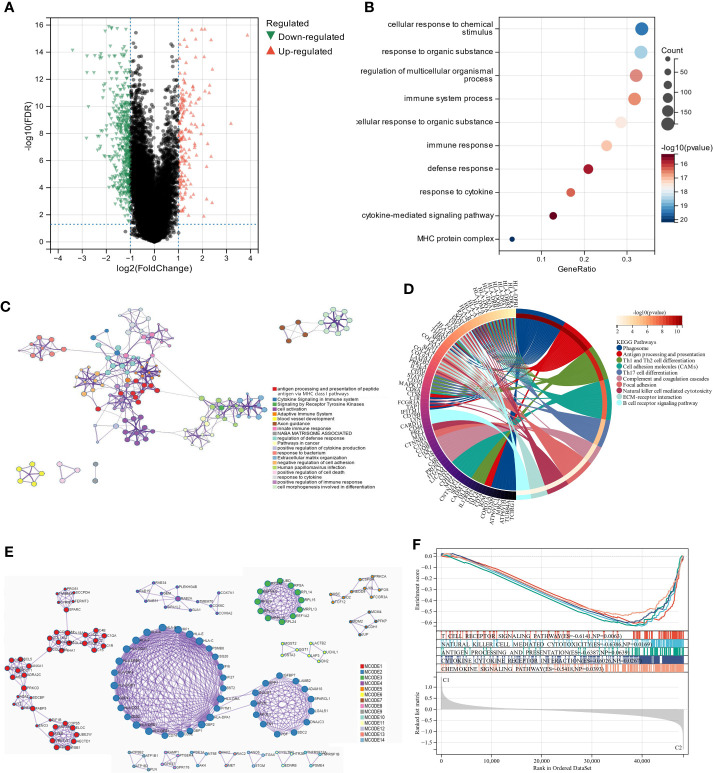
The analysis of differentially expressed genes and the evaluation of their functions. **(A)** Volcano plot illustrates the expression of DEGs between the two subgroups. **(B, C)** Visualization of biological processes that have been enhanced by GO analysis using bubble diagrams and networks. **(D)** A circle plot displaying the signaling pathways that KEGG analysis has enriched. **(E)** An analysis of DEGs based on PPI. **(F)** A GSEA plot depicts the signaling pathway analysis.

We used the “limma” R tool to compare gene expression between groups. The two clusters had 647 DEGs, 170 upregulated and 477 downregulated (**Figure 3A**). To elucidate these DEGs’ immunity-related functions, GO (**Figures 3B, C**) and KEGG (**Figure 3D**) analyses were conducted. Most of these DEGs were involved in antigen processing and presentation, Th1 and Th2 cell differentiation, and other immune-related functions, according to signaling pathway analyses. The PPI analysis identified 14 sub-models, most of which (MCODE1, 2, 5, 7, 8, 10, and 12) were closely related to tumor formation and immunity, suggesting that immunity may contribute to UM through lipid metabolism (**Figure 3E**; **Table S5**). GSEA was performed in the two clusters to determine the relationship between enriched pathways and immune cell infiltration in UM patients, which found that T cell receptor signaling pathway, natural killer cell-mediated cytotoxicity, antigen processing and presentation, cytokine-cytokine receptor interaction, and chemokine signaling pathways expressed highly in cluster 1 patients (**Figure 3F**). These findings suggest that LMRG expression is crucial in the UM TME.

### 3.3 UM patients in two molecular subtypes exhibited significant differences in TME and immune status

**Figure 4:**
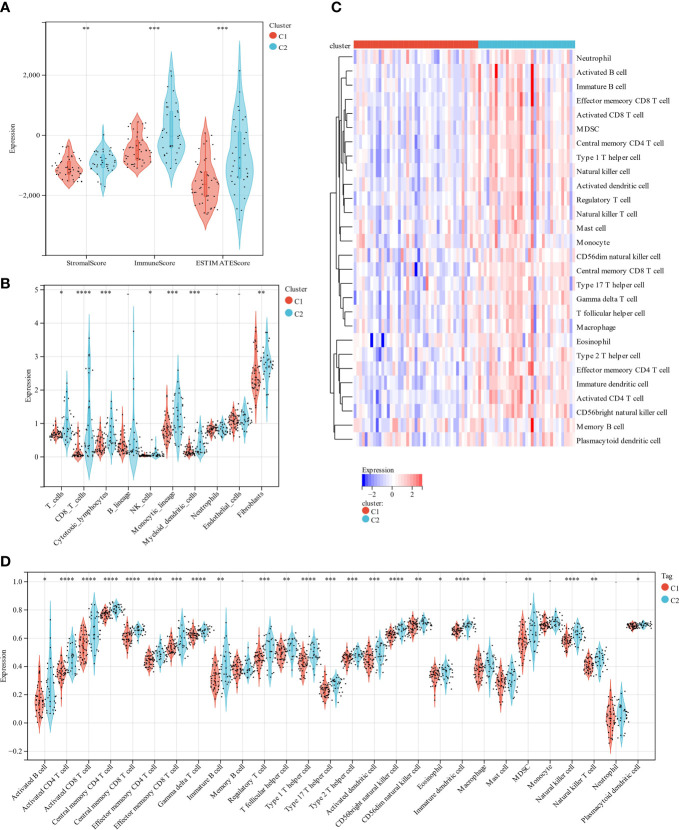
A comparison of immune infiltration between the two clustered subgroups. **(A)** The Violin diagram presented the calculation of stromal score, immune score, and estimate score in two subgroups. **(B)** MCP evaluated the abundance of ten immune filtrating cells. **(C)** A heatmap displays the level of enrichment for 28 immune-related cells based on a ssGSEA algorithm. **(D)** Statistical analysis of ssGSEA. *p < 0.05; **p < 0.01; ***p < 0.001 ****p < 0.0001.

The scoring signature of tumor-infiltrated immune cells can predict immunological treatment response and UM prognosis (42). Thus, we identify the relationship between lipid metabolism and TME in UM. We calculated each subgroup’s immune score, ESTIMATE score, and stromal score using the ESTIMATE technique to see if there was an immunological difference. Cluster 2 showed much higher immune scores than cluster 1 (**Figure 4A**). Furthermore, we estimated immune infiltration in the UM microenvironment using the TIMER database. Cluster 1 had more fibroblasts, but cluster 2 had more T cells, CD8 T cells, cytotoxic lymphocytes, NK cells, monocytic lineage, and myeloid dendritic cells (**Figure 4B**). B lineage, neutrophils, and endothelial cells were not statistically different between these two clusters (**Figure 4B**). Cluster 1 had a relatively low immune status, while cluster 2 was high (**Figures 4C, D**). These findings suggest that two molecular subtypes have distinct TME.

### 3.4 Construction of a risk model

**Figure 5:**
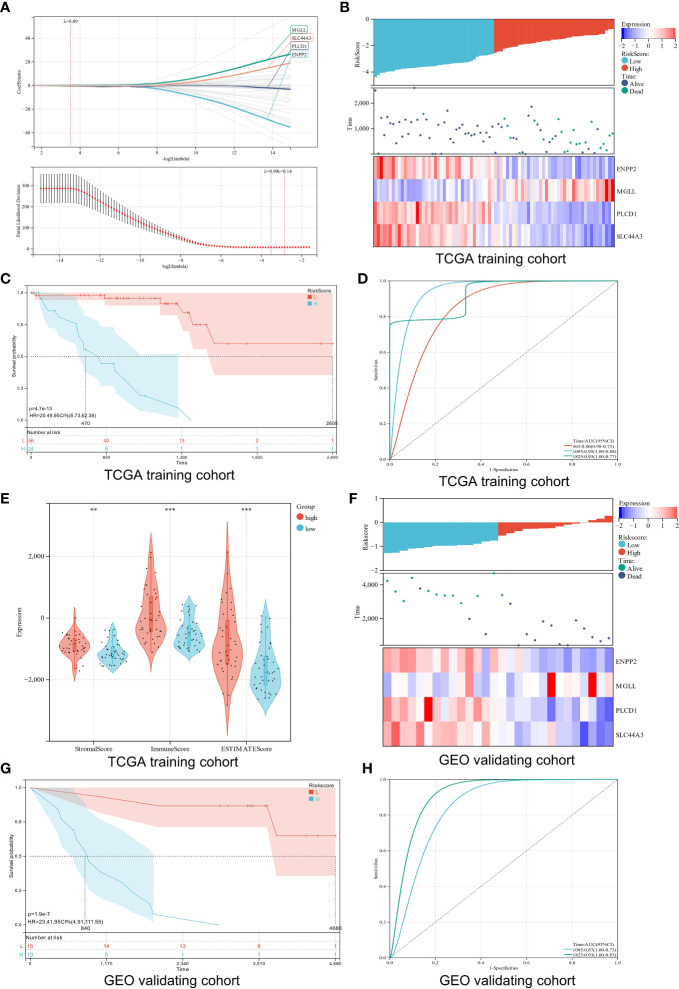
Construction and verification of a risk model. **(A)** Analysis using LASSO with a minimal lambda. **(B)** A heatmap displays the survival status and risk score of UM patients based on the expression of four potential genes in two groups. **(C)** Survival curves of UM patients in two different groups. **(D)** A risk model with ROC curves that are time-dependent. **(E)** Stomal score, immune score, and ESTIMATE score are calculated using the ESTIMATE algorithm. **(F)** Four candidate genes were expressed in the verification cohort with survival status and risk score indicated. **(G)** An analysis of the survival curves for high-risk and low-risk patients in the verification cohort. **(H)** The ROC curve for the risk model’s verification cohort. **p < 0.01; ***p < 0.001

A prognosis prediction model was used to test LMRGs’ ability to predict UM prognosis. We extracted four LMRGs in UM] at the minimum likelihood of a deviative pattern (λ_min_ = 0.09) using Lasso regression on these DEGs (**Figures 5A, B**). Four genes (ENPP2, MGLL, PLCD1, and SLC44A3) were discovered through multivariate Cox regression. These four genes were utilized to create a risk regression model that categorized UM patients into low- and high-risk groups (**Figure 5C**). Patients with high risk exhibited significantly shorter survival periods than those with low risk (P = 4.7e-13; **Figure 5D**). The model’s robustness was assessed by plotting ROC curves for 1-year, 3-year, and 5-year OS, with areas under curves (AUCs) of 0.86, 0.95, and 0.93, respectively (**Figure 5D**), indicating a positive accuracy rate. We used the ESTIMATE algorithm to evaluate the two groups’ TME to better understand TME involvement in the UM risk model. The high-risk group had significantly higher stromal, immune, and ESTIMATE scores than the low-risk group (**Figure 5E**). Generally, high immune cell infiltration in the TME is associated with a positive prognosis, but in the UM it is associated with poor outcomes (42), which is consistent with our findings.

Additionally, the verification cohort was used to validate the created predictive risk score model. The heatmap showed the four candidate genes expression in the verification cohort stratified by risk level (**Figure 5F**). Survival analysis showed that the high-risk group had a worse prognosis than the low-risk group (P = 1.9e-7; **Figure 5G**). ROC analysis showed that the risk model predicted 5-year survival best (**Figure 5H**). Our findings show that the risk score model we created can correctly forecast the prognosis of UM patients.

### 3.5 Testing for independence in the constructed risk model

Subgroup analysis and regression analysis were used to evaluate the risk model’s independence and the risk score’s relevance to clinical features. Risk scores did not differ between patients of different sexes (**Figure S1A**), ages (**Figure S1B**), or distant metastases (**Figure S1C**), indicating no correlation between risk scores and clinical characteristics. The risk model was highly predictive when patients were classified by sex (**Figures S1D, E**), age (**Figures S1F, G**), or distant metastasis (**Figures S1H, I**). The risk model was also an independent predictor of patient prognosis in the univariate Cox regression study (**Table 1**). These findings demonstrate that the risk model is extremely independent in predicting UM patients’ prognoses.

**Table 1:** Univariate analysis of risk score and characteristics in training cohort.

Characteristics	Total (N)	HR (95% CI) Univariate analysis	P value Univariate analysis	HR (95% CI) Multivariate analysis	P value Multivariate analysis
Age	80	1.046 (1.008 - 1.085)	< 0.05	1.092 (1.019 - 1.171)	< 0.05
Gender	80		0.316		
Male	45	Reference			
Female	35	0.649 (0.274 - 1.536)	0.325		
Clinical T stage	80		0.169		
T2	5	Reference			
T3	36	0.956 (0.116 - 7.864)	0.966		
T4	39	2.163 (0.278 - 16.817)	0.461		
Clinical N stage	80		0.058		
N0	76	Reference		Reference	
NX	4	6.177 (1.302 - 29.304)	< 0.05	8.059 (0.560 - 115.918)	0.125
Clinical M stage	80		< 0.01		
M0	73	Reference		Reference	
M1	3	35.072 (4.689 - 262.335)	< 0.001	0.241 (0.006 - 9.795)	0.452
MX	4	2.226 (0.507 - 9.778)	0.289	0.694 (0.051 - 9.367)	0.783
Clinical stage	80		< 0.001		
Stage II	39	Reference		Reference	
Stage III	37	1.235 (0.504 - 3.028)	0.644	1.074 (0.318 - 3.623)	0.909
Stage IV	4	72.950 (7.056 - 754.174)	< 0.001	20.228 (0.383 - 1066.974)	0.137
Metastasis	80		< 0.001		
Yes	26	Reference		Reference	
No	54	0.044 (0.010 - 0.189)	< 0.001	0.118 (0.015 - 0.956)	< 0.05
RiskScore	80	36.695 (8.799 - 153.035)	< 0.001	12.017 (1.637 - 88.233)	< 0.05

HR, hazard ratio; CI, confidence interval.

### 3.6 Construction and calibration of an integrated nomogram combining clinicopathological features and risk signature

Clinical parameters should be considered while predicting UM patients’ prognoses, therefore risk score, age, gender, and distant metastases were used to create a prognosis nomogram for UM patients (**Figure S2A**). The nomogram was validated in the training and verification cohorts using the concordance index (C-index) and calibration curve. The nomogram’s C‐index in the training group was 0.919 (95% CI, 0.887–0.951, **Figure S2B**), which matched the verification cohort (**Figure S2C**), indicating its predictive power. Overall, the nomogram was more accurate in predicting UM patients’ prognosis.

### 3.7 MGLL affects the proliferation, migration, and invasion of UM cells *in vitro*

**Figure 6:**
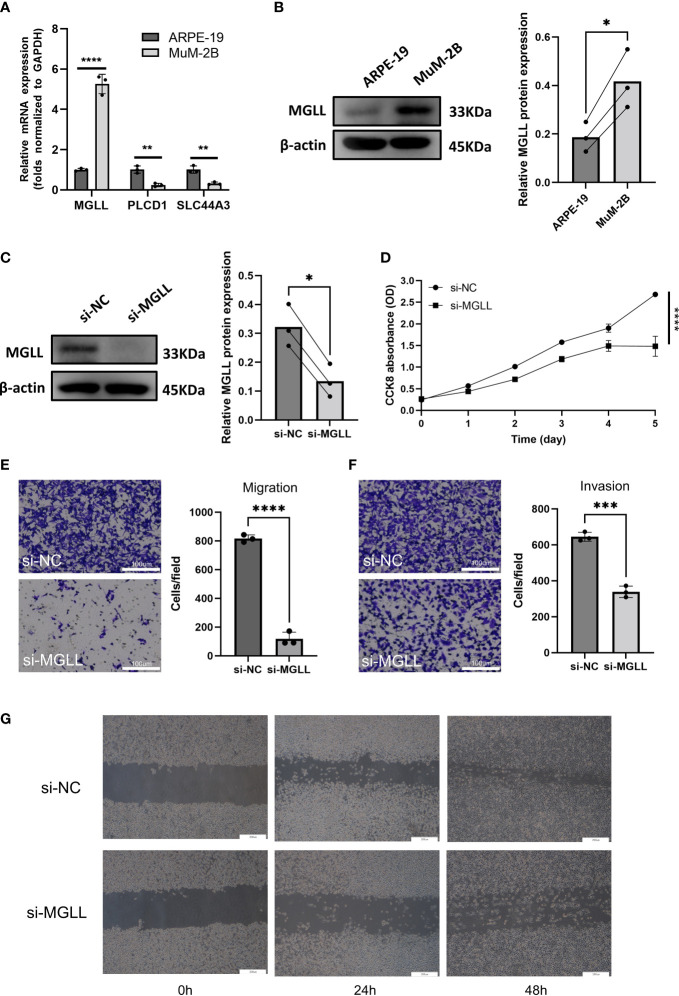
MGLL knockdown reduces UM cell migration, invasion, and activity *in vitro*. **(A)** Using qRT-PCR, the expression levels of ENPP2, MGLL, PLCD1, and SLC44A3 in ARPE-19 and MuM-2B cells were determined. ENPP2 mRNA expression was undetectable. Normalization of Ct values to GAPDH was performed. (Student’s t-test) **(B)** Using WB, MGLL levels in ARPE-19 and MuM-2B cells were examined, and relative protein expression was adjusted using -actin levels. (left: images indicative of three independent experiments; right: quantitative analysis, n = 3, paired Student’s t-test) **(C)** Western blotting revealed MGLL protein level in response to MGLL-siRNA treatment; relative protein expression levels were normalized based on -actin levels. (left: photos indicative of three independent experiments; right: quantitative analysis, n = 3, paired Student’s t-test) **(D)** Using the CCK-8 test, growth curves for MuM-2B cells treated with MGLL knockdown were determined. (Student’s t-test) **(E)** To determine the migration of MuM-2B cells, a Transwell test was carried out. (left: typical images from three independent experiments; right: quantitative analysis, n = 3, unpaired Student’s t-test, scale bar represents 100 μm) **(F)** A Transwell experiment was conducted to identify the invasion of MuM-2B cells following MGLL knockdown treatment. (left: typical images from three independent experiments; right: quantitative analysis, n = 3, unpaired Student’s t-test, scale bar represents 100 μm) **(G)** MuM-2B cells were used in a wound healing experiment to identify MGLL knockdown-induced migration. (typical images of three independent experiments, scale bar represents 200 μm) *p < 0.05; **p < 0.01; ***p < 0.001; ****p < 0.0001.

When ENPP2, MGLL, PLCD1, and SLC44A3 mRNA expression levels in ARPE-19 cells and MuM-2B cells were compared, we found that MGLL had significantly higher expression while PLCD1 and SLC44A3 had significantly lower expression (**Figure 6A**). ENPP2 mRNA expression was not detectable in this study. MuM-2B cells were found to have much higher expression levels of MGLL than ARPE-19 cells (**Figure 6B**, **Figure S3**). siRNA-targeted MGLL and siRNA control were transfected into MuM-2B cells to explore the functional role of MGLL in UM. Transfection effectiveness was assessed using Western blotting (**Figures 6C**, **S4**). The impact of MGLL on cell proliferation was then assessed using the CCK-8 test, which revealed that MGLL knockdown reduced the proliferation of MuM-2B cells (**Figure 6D**). Additionally, after reducing the expression of MGLL, MuM-2B cells’ capacity for migration and invasion was suppressed (**Figures 6E–G**, **S5–S7**). According to these findings, MGLL inhibition reduced cell proliferation and migration in UM.

### 3.8 Pan-cancer MGLL expression and prognosis

**Figure 7:**
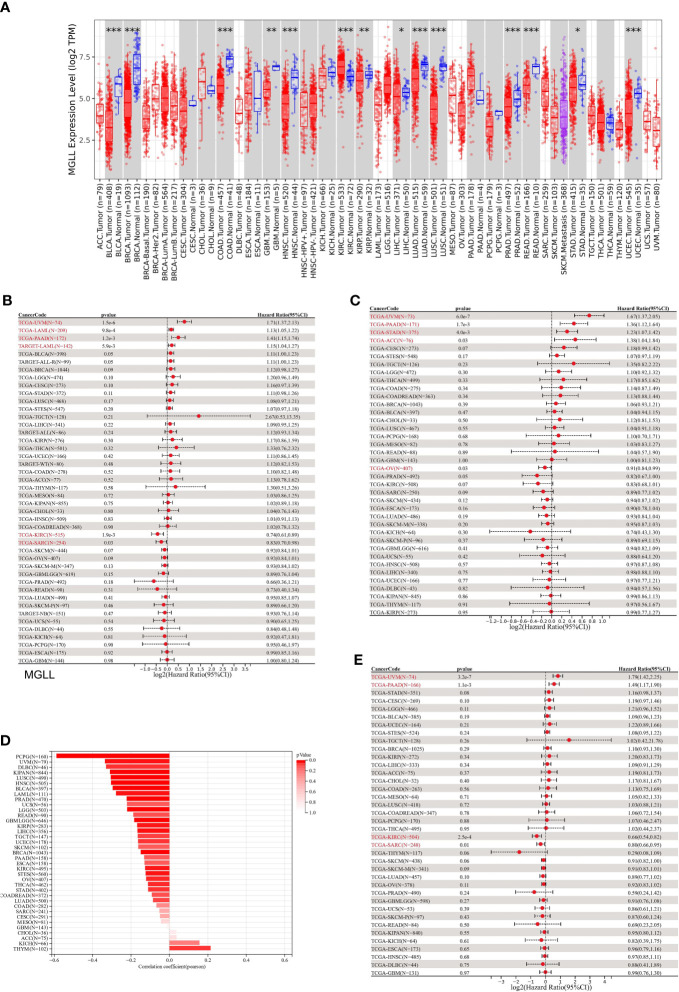
A correlation between MGLL and prognosis in pan-cancer samples. **(A)** Expression levels of MGLL across pan-cancer samples in the TCGA dataset. (http://timer.cistrome.org/) **(B)** Cox regression model study of MGLL expression and OS in the UCSC dataset. **(C)** Cox regression model-based analysis of PFS and MGLL expression. **(D)** Cox regression analysis of MGLL expression with DSS in different types of tumor. **(E)** MGLL expression and tumor purity were correlated using the ESTIMATE algorithm in the UCSC dataset. DSS, disease-specific survival; PFS, progression-free survival. *p < 0.05; **p < 0.01; ***p < 0.001.

Differential expression analysis of pan-cancer samples revealed that MGLL was generally underexpressed in cancers, including bladder urothelial carcinoma (BLCA, p < 0.001), breast invasive carcinoma (BRCA, p < 0.001), colon adenocarcinoma (COAD, p < 0.001), glioblastoma multiforme (GBM, p < 0.01), head and neck squamous cell carcinoma (HNSC, p < 0.001), kidney renal papillary cell carcinoma (KIRP, p < 0.01), liver hepatocellular carcinoma (LIHC, p < 0.05), lung adenocarcinoma (LUAD, p < 0.001), lung squamous cell carcinoma (LUSC, p < 0.05), prostate adenocarcinoma (PRAD, p < 0.001), rectum adenocarcinoma (READ, p < 0.05), stomach adenocarcinoma (STAD, p < 0.05), and uterine corpus endometrial carcinoma (UCEC, p < 0.001), with the exception of kidney renal clear cell carcinoma (KIRC, p < 0.001) (**Figure 7A**).

MGLL expression was then evaluated in relation to OS, DSS, and PFS. An analysis of 44 tumors using cox regression revealed that MGLL expression was significantly positively related to the OS of UM patients (p = 1.5e-6), acute myeloid leukemia patients (LAML, p = 9.8e-4 in TCGA, p = 5.9e-3 in TARGET), and pancreatic adenocarcinoma patients (PAAD, p = 1.2e-3), but negatively related to the OS of KIRC patients (p = 1.9e-3) and sarcoma patients (SARC, p = 0.03) patients (**Figure 7B**). Further cox regression analysis of 38 tumors indicated that MGLL expression significantly correlated with PFS in 5 cancers and was a risk factor for UM (p = 6.0e-7), PAAD (p = 1.7e-3), STAD (p = 4.0e-3), and ACC (p = 0.03), but a protective factor for ovarian serous cystadenocarcinoma (OV, p=0.03; **Figure 7C**). MGLL expression was significantly correlated with DSS in four tumors. For UM (p = 3.2e-7) and PAAD patients (p = 1.1e-3), MGLL was a protective factor, whereas for KIRC (p = 2.5e-4) and SARC patients (p = 0.01), it was a risk factor (**Figure 7D**).

Tumor tissues contain many non-tumor cells, including immune cells, stromal cells, and interstitial cells, which help tumor formation and growth (32). Tumor purity correlated with clinical characteristics, genome expression, and characteristics (43). Thus, MGLL expression and tumor purity should be assessed in tumor samples (**Figure 7E**). 22 tumors had significant Pearson correlations, 1 of which was positive and 21 of which were negative (**Table S6**).

### 3.9 Immune infiltration analysis

**Figure 8:**
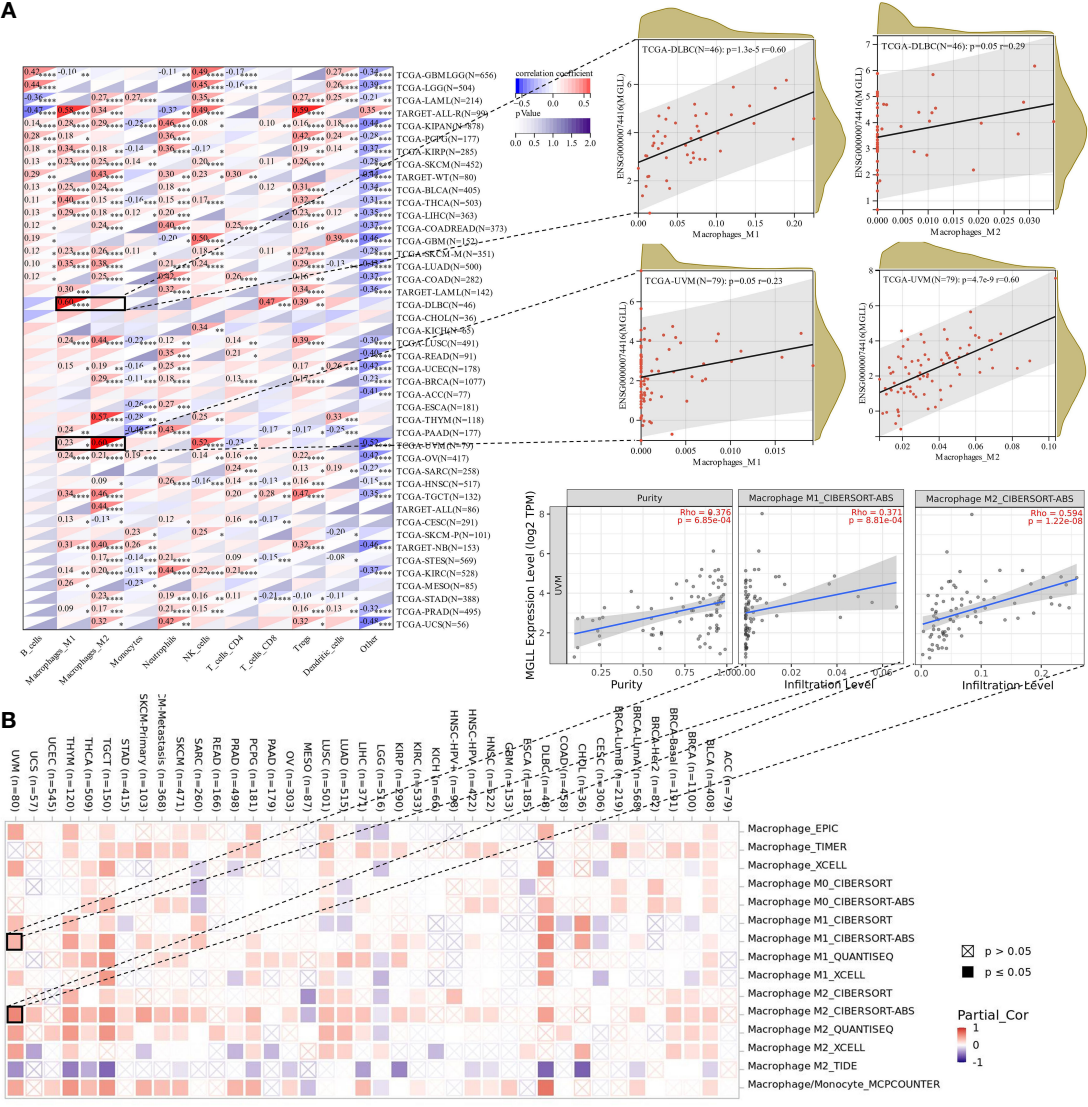
Assessment of immune infiltration. **(A)** Using the QUANTISEQ method, MGLL expression and immune cell infiltration were associated in the UCSC dataset. **(B)** Various algorithms identifies a relationship between MGLL expression and macrophages in different types of cancer from the TCGA database. *p < 0.05; **p < 0.01; ***p < 0.001; ****p < 0.0001.

Since this work indicated a relationship between MGLL expression and immune cell infiltration in various malignancies (**Figure 7E**), we explored immune cell infiltration in pan-cancers. QUANTISEQ analyzed the immune cell infiltration of a uniformly normalized dataset from the UCSC database. MGLL and immune cell infiltration scores were strongly associated with 10,180 tumor samples from 44 tumor types. Besides, MGLL expression and immune infiltration were correlated in 43 cancer types (**Figure 8A**). Interestingly, MGLL gene expression was highly positively related to M1-type macrophages in acute lymphoblastic leukemia (ALL, R = 0.58) and lymphoid neoplasm diffuse large B-cell lymphoma (DLBC, R =0.60; **Figure 8A**) and considerably positively associated with M2-type macrophages in UM (R = 0.60; **Figure 8A**).

Using TIMER2, we examined the connection between pan-cancer macrophage infiltration and MGLL expression and found a positive correlation between them in DLBC, testicular germ cell tumor (TGCT), thymoma (THYM), and UM (**Figure 8B**). CIBERSORT algorithm also revealed a favorable association between UM MGLL expression and M2 macrophages (R = 0.59; **Figure 8B**). Therefore, MGLL affects immune cell infiltration especially macrophage polarization in various cancers.

### 3.10 Effect of MGLL on macrophage infiltration in TME

**Figure 9:**
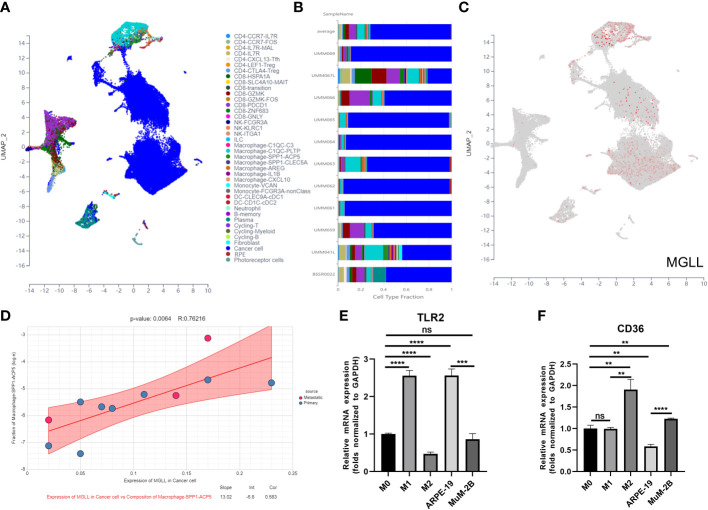
MGLL affects macrophage polarization in the TME. **(A)** Distribution of different cell clusters in the UMAP plot. **(B)** The fraction of each sample depends on the type of cell. **(C)** MGLL expression in distinct clusters of cells. **(D)** Correlation analysis between expression of MGLL in cancer cells and composition of infiltrating macrophages (SPP1-ACP5). **(E)** THP-1 macrophages were polarized towards an M1-like phenotype by the conditioned media of ARPE-19 cells with reduced MGLL expression. **(F)** THP-1 macrophages were polarized to an M2-like phenotype by the conditioned medium of MuM-2B cells with highly expressed MGLL. (n = 3, paired Student’s t-test) **p < 0.01; ***p < 0.001; ****p < 0.0001 ns, no significance.

We used Uniform Manifold Approximation and Projection (UMAP) to cluster and designate 59,916 cells into 40 categories utilizing UM single-cell sequencing data (GSE139829) to study MGLL expression and function in TME at single-cell resolution (**Figure 9A**). Each sample’s cell type fraction, with cancer cells, makes up practically the entire part (**Figure 9B**). Cancer cells and macrophages predominately expressed MGLL (**Figure 9C**). We then examined MGLL expression in cancer cells and macrophage makeup invading them (**Figures 9D**, **S8A–F**) and found that MGLL expression on cancer cells was positively correlated with SPP1-ACP5 macrophage infiltration (**Figure 9D**). SPP1-ACP5 macrophages were confirmed using the gene markers TLR2 of macrophage M1 and CD36 of M2. SPP1-ACP5 and IL1B macrophages express high levels of CD36 (**Figure S8G**), while ARG and IL1B macrophages express high levels of TLR2 (**Figure S8H**). Interestingly, MGLL may polarize M2 macrophages (SPP1-ACP5) by eliminating IL1B macrophages (**Figure 9D**).

To test if tumor cell-expressed MGLL can polarize macrophages to the M2 type, ARPE-19 or MuM-2B cells were co-cultured with THP-1 cells. Co-culturing MuM-2B and THP-1 cells elevated M2 macrophage markers including CD36 (**Figure 9F**), but not M1 markers like TLR2 (**Figure 9E**). In contrast, ARPE-19 cells co-cultured with THP-1 cells showed opposing macrophage polarization. These data suggest that UM-produced MGLL regulates macrophage polarization.

## 4 Discussion

Numerous lines of evidence imply that lipid metabolism is reprogrammed in cancers (44), which contributes to tumor progression and local immunosuppression in the TME (26). Lipids, cell membrane components and second messengers that transduce signals within cells serve as vital energy storage sources under nutrient scarcity (45). The function of lipid metabolic abnormalities in cancer cells has been a major topic of study in recent years. The key processes of lipid metabolism are synthesis, storage, and breakdown. There is evidence that aberrant lipid metabolism plays a significant role in the development, progression, invasion, and treatment response of numerous cancers (46).

UM, the most common primary malignant eye tumor has been a major public health issue. Although UM will be diagnosed earlier as diagnostic technology advances, a fraction of early-stage patients are still diagnosed at an advanced stage, and the 5-year survival rate is still dismal, with the median survival of metastatic UM patients being less than 1 year due to high metastasis rates and restricted therapy options (3, 47, 48). Thus, better risk stratification strategies are needed to identify high-risk cancer patients to improve their prognosis.

Using consensus clustering, we classified samples into two categories based on the mRNA expression patterns of 47 prognostic genes derived from univariable Cox analysis. We found that lipid metabolism abnormalities may affect patient outcomes, as the two molecular categories had significant differences in overall survival, which we speculated may be linked to immune activity. Therefore, the ESTIMATE algorithm was then used to give additional insight into the immunological landscapes of UM, revealing that UM patients with bad prognoses had higher immune scores and ESTIMATE scores than those with better prognoses. Based on the aforementioned data, it can be assumed that immune variants may have a significant role in UM survival.

Furthermore, functional investigations were conducted to investigate the underlying mechanisms. In this study, we demonstrated that the prognosis of UM is significantly influenced by LMAGs through immune-associated signaling pathways, per the GO analysis and KEGG analysis. Subsequently, GSEA was used to explore the association between lipid metabolism and aberrant immunity. The findings showed that cluster 2 had lower immune cell differentiation expression. These findings provided a preliminary explanation for the prognostic differences between the two groupings, showing that immunological activity and the associated-LMRGs were responsible.

Additionally, we constructed a predictive risk model based on LMRGs and verified it in a validation cohort to establish that lipid metabolic disorders affect TME in UM patients. We also constructed a prognostic risk model using the four LMRGs signatures, including ENPP2, MGLL, PLCD1, and SLC44A3, and we found that most of them were correlated with tumor progression. For example, ENPP2, which encodes autotaxin, is overexpressed in chronic inflammatory diseases and cancer and synthesizes lysophosphatidic acid (49, 50). PLCD1 is known to convert phosphatidylinositol bisphosphate into diacylglycerol and inositol triphosphate, which serve as scaffolds and signaling molecules (51). PLCD1 is also identified as a new tumor suppressor gene, which is suppressed by promoter methylation in various cancer types (51–53). However, SLC44A3’s role in cancer is unknown. Notably, only MGLL was highly expressed in the risk model. MGLL is a metabolic enzyme that transforms triglycerides into free fatty acids and is involved in tumor signaling (54). MGLL has been implicated to play a pathophysiological role in various cancers (54, 55). MALL is found to be involved in multiple cellular processes in cancer cells and is highly elevated in multiple aggressive cancer types (56). For instance, in endometrial adenocarcinoma, MGLL promoted tumor proliferation, metastasis, and the occurrence of progestogen resistance (57). In lung cancer, MGLL inhibition led to a decrease in cell proliferation, invasion, and metastasis (58, 59). In addition to inhibiting cell proliferation, migration, invasion, and tumor growth, MAGL inhibition also induced apoptosis in cervical cancer (60). According to a study on TNBC suggested that inhibiting MGLL can suppress inflammation, tumor growth, and brain colonization (61). Interestingly, MGLL in cancer cells promoted tumor progression by releasing special fatty acids whereas MGLL in TAMs suppressed cancer development by attenuating endogenous cannabinoid receptor 2 signaling (62). Additionally, MGLL has been reported to play a role in melanoma. Baba et al. observed that melanoma samples with lymphovascular invasion tended to be expressed more MGLL than samples without invasion, suggesting that the expression of MGLL in tumor cells may serve as a marker of tumor invasion and progression in malignant melanoma (63). In this study, we found that MGLL is upregulated in UM, and inhibition of MGLL suppressed the cell proliferation, migration, and invasion of UM cells, suggesting an oncogenic role of MGLL in UM.

Lipid metabolism has a substantial impact on macrophage regulatory functions. For example, lipids not only supply energy but also provide precursors to bioactive lipids and cell membrane components to macrophage (64). Besides, lipids regulate gene expression and signal transduction during macrophage activation (65). Several studies revealed that MGLL can induce the accumulation of 2-arachidonoylglycerol in the TME, which promotes the shift of tumor-associated macrophages into a tumor-promoting M2-like state by activating CB-2 (56, 62). Similarly, we discovered that MGLL was abundantly expressed in UM cells, which prompted a phenotypic shift in macrophages to a pro-tumor M2 state. Therefore, inhibiting MGLL in tumor cells may be a curative treatment for UM.

The 4-LMRGs risk model assigned a risk score to each UM patient, and the examination of survival in both the training and validation cohorts demonstrated powerful prediction ability. Using univariate/multivariate Cox regression analysis, the built risk model was an independent predictor of prognosis for UM patients irrespective of age, sex, or metastatic status. Additionally, we created and validated a nomogram that combines risk ratings and clinical characteristics to predict survival. The results indicated that aberrant lipid metabolism and TME may have an effect on therapy and survival, particularly in patients with metastatic UM.

Although our established LMRGs-based risk score for the prognosis of UM patients showed potential, there were disadvantages to being notified in our study. An initial disadvantage of the study is that differences in demographic variables, such as race, lifestyle, and living conditions were not taken into account. Second, our result was derived from open databases and not our cohorts’ data. Thirdly, *in vivo* experiments are needed to further validate our results.

In conclusion, we discovered two genetic subgroups based on LMRGs in UM and assessed the significance of LMAGs in patients’ prognosis and immune microenvironment. In addition, the molecular mechanisms may include the deregulation of lipid metabolism, which impedes the immune system and contributes to a bad prognosis. Additionally, we found that the actions of LMAGs in UM may be mediated by immune-related signaling pathways. We also discovered that cancer cells had elevated levels of MGLL expression, which switched macrophages to the pro-tumor M2 phenotype. Our work may shed light on the creation of new targeted medications and gives a potential direction for future UM research and personalized therapy.

## Data availability statement

The original contributions presented in the study are included in the article/**Supplementary Material**. Further inquiries can be directed to the corresponding authors.

## Author contributions

YT and BQ conceived the study. YT and ZD collected and cleaned the data. YT and JP wrote the original draft. YT, JP, and ZL participated in completing the experiment. TC and JX participated in the formal analysis and data curation. ZD, CZ, and BQ revised the article. All authors contributed to the article and approved the submitted version.
