# Supplementary material for: Monoacylglycerol lipase regulates macrophage polarization and cancer progression in uveal melanoma and pan-cancer
Source: Front Immunol. 2023 Mar 23;14:1161960. doi: 10.3389/fimmu.2023.1161960 (PMC10076602; doi:10.3389/fimmu.2023.1161960)
Supplement: Supplementary file 1 [file DataSheet_1.docx]

Supplementary Material

# Supplementary Figures and Tables

##
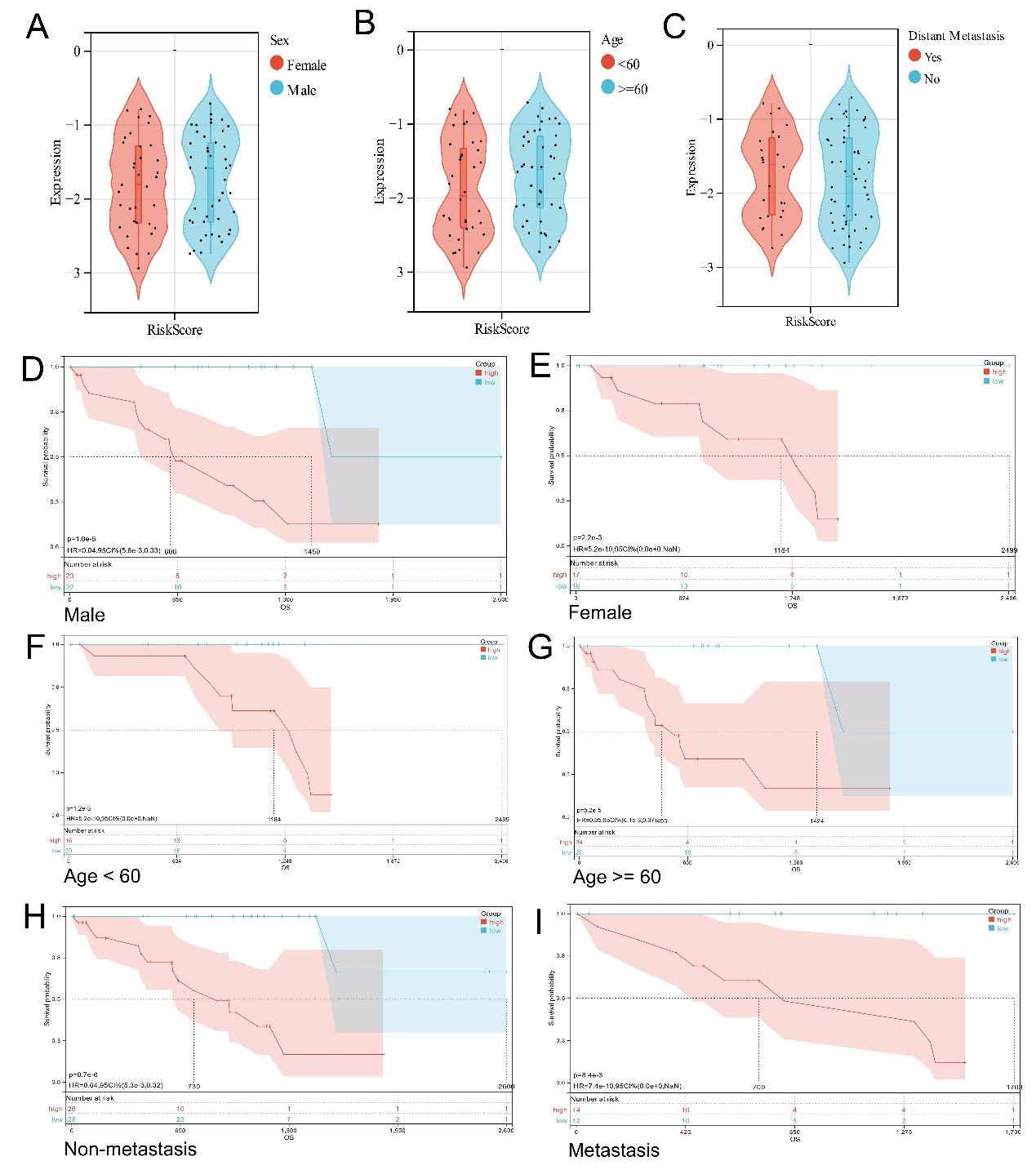
Supplementary Figures

Supplementary Figure 1 | Independence of the constructed risk model. An analysis of the association between risk scores and clinical characteristics (A–C). In patients of different sex (A), age (B), and metastasis status (C), no significant differences were found. An analysis of the independence of the risk model (D-I). A survival curve showing the survival rates of patients regrouped according to their sex (D, male; E, female), age (F, <60; G, >=60), and metastasis (H, non-metastasis; I, metastasis).


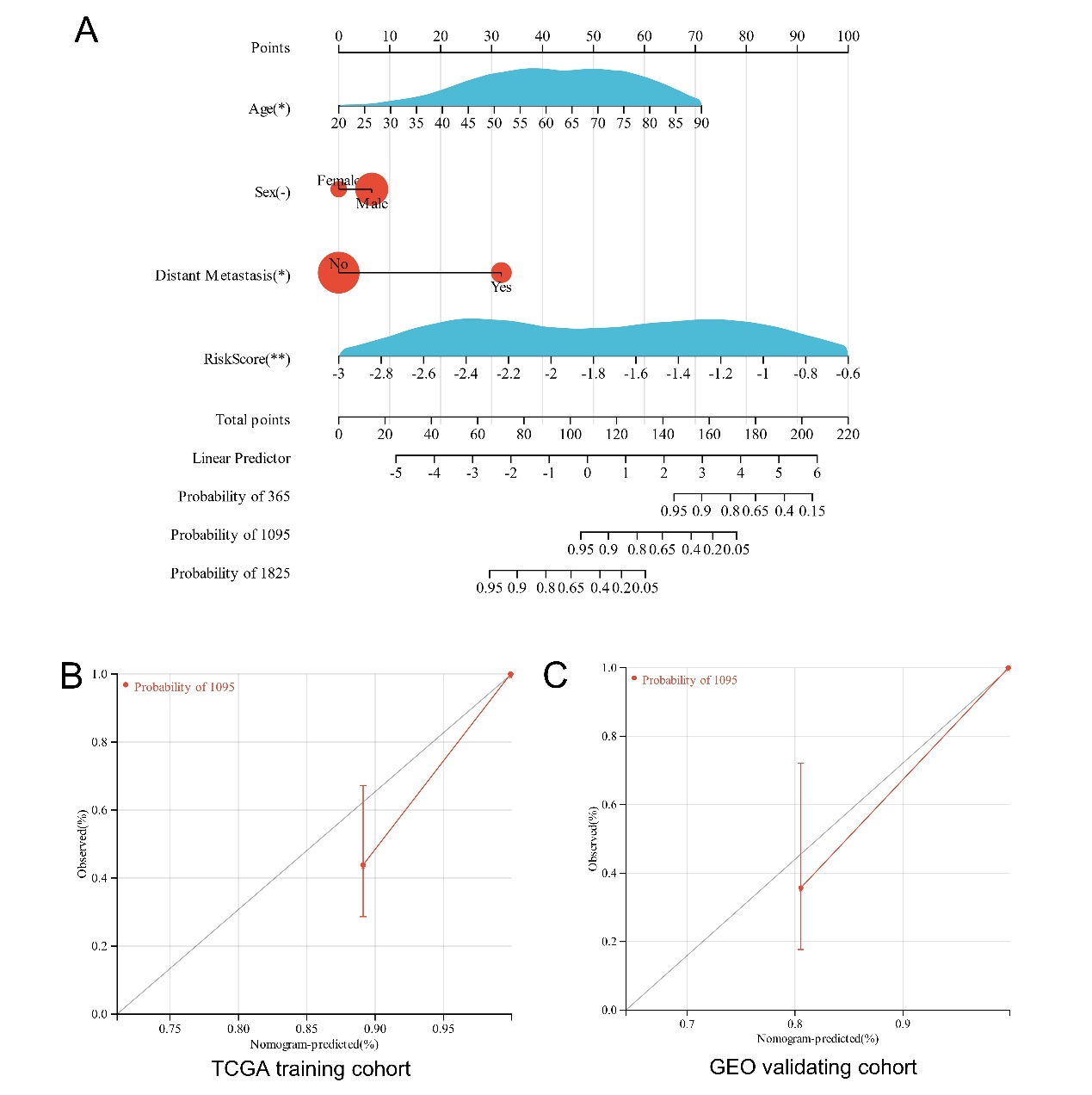


Supplementary Figure 2 | Nomogram construction and calibration. (A) Nomogram integrating clinical and risk factors. (B) The nomogram was calibrated at 3 years in the training cohort and the validation cohort.


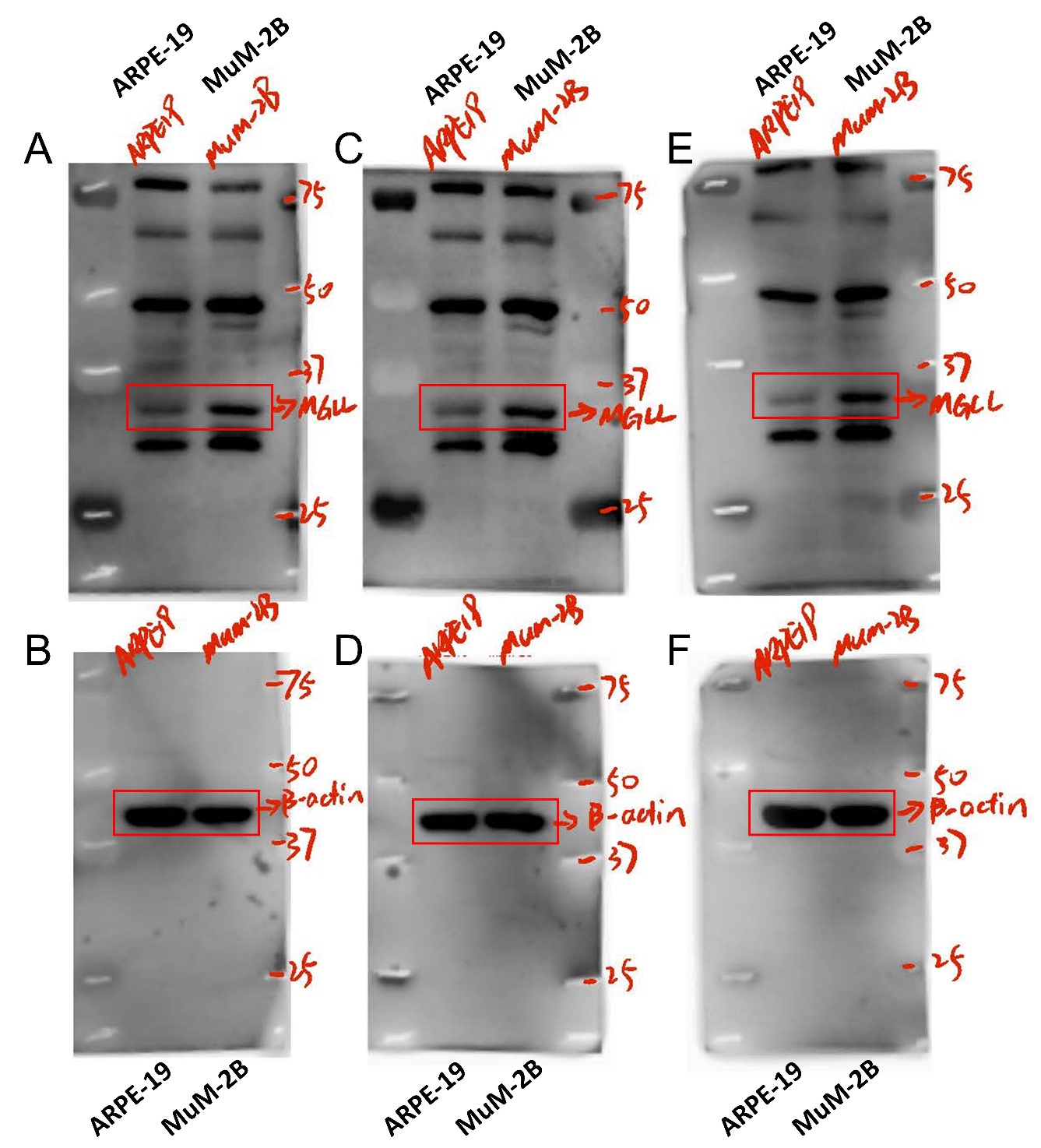
Supplementary Figure 3 | Full immunoblots of images presented in the manuscript. The membrane was probed for MGLL (A, C, E) and re-probed for β-actin (B, D, F) in ARPE-19 and MuM-2B cells. A-B, C-D, E-F are three independent experiments. All gels are 10.0%. All PVDF membranes are full membranes without trimming. And the bands will be washed with stripping buffer to remove previous antibody and imaged again. Red boxes mark specific bands. Outside the red frame are marks non-specific bands.


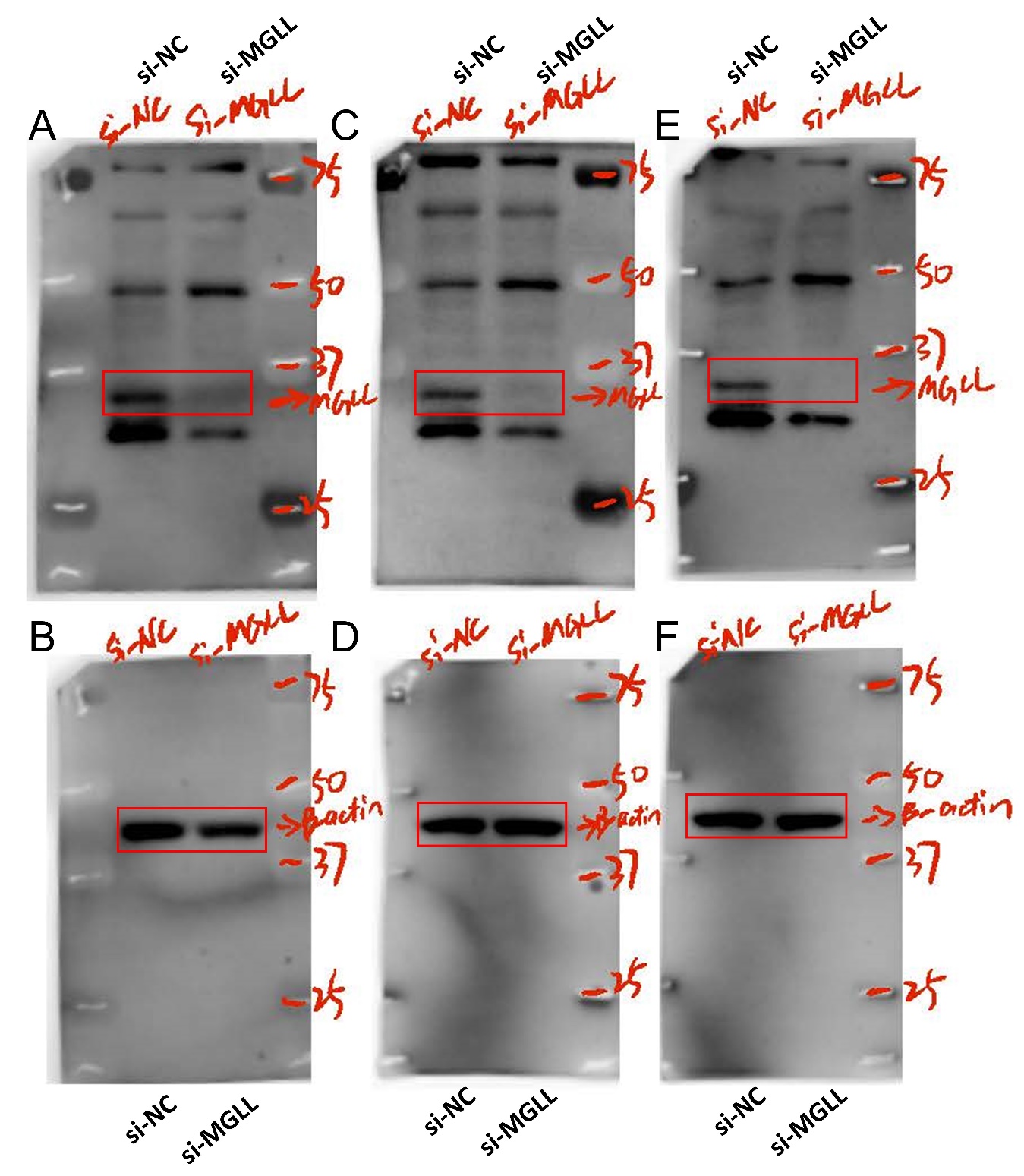
Supplementary Figure 4 | Full immunoblots of images presented in the manuscript. The membrane was probed for MGLL (A, C, E) and re-probed for β-actin (B, D, F) in control and MGLL-siRNA treatment. A-B, C-D, E-F are three independent experiments. All gels are 10.0%. All PVDF membranes are full membranes without trimming. And the bands will be washed with stripping buffer to remove previous antibody and imaged again. Red boxes mark specific bands. Outside the red frame are marks non-specific bands.


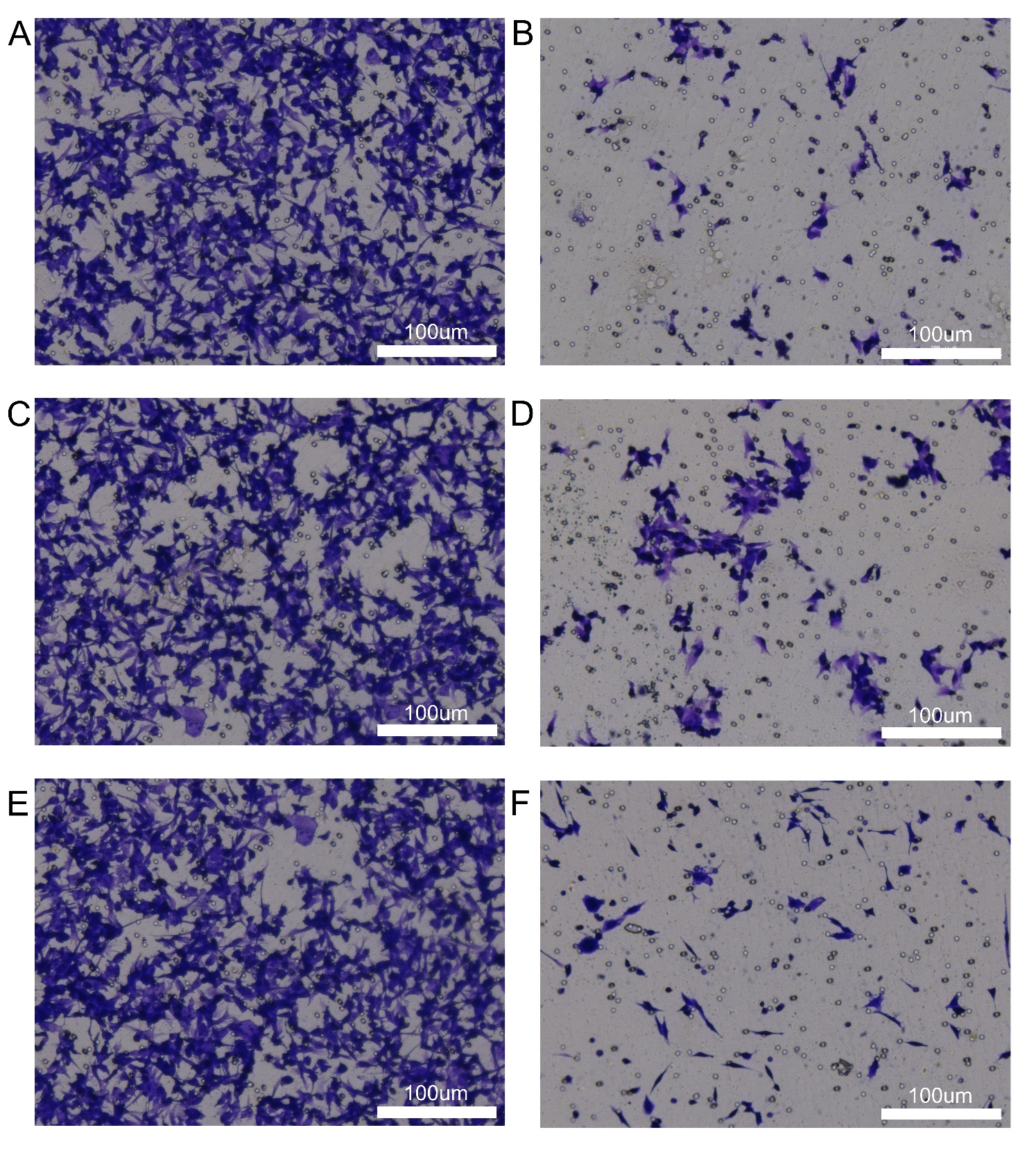
Supplementary Figure 5 | Transwell assay was used to examine the cell migration ability of si-NC (A, C, E) and si-MGLL (B, D, F) UM cells. A-B, C-D, E-F are three independent experiments. Scale bar represents 100 μm.


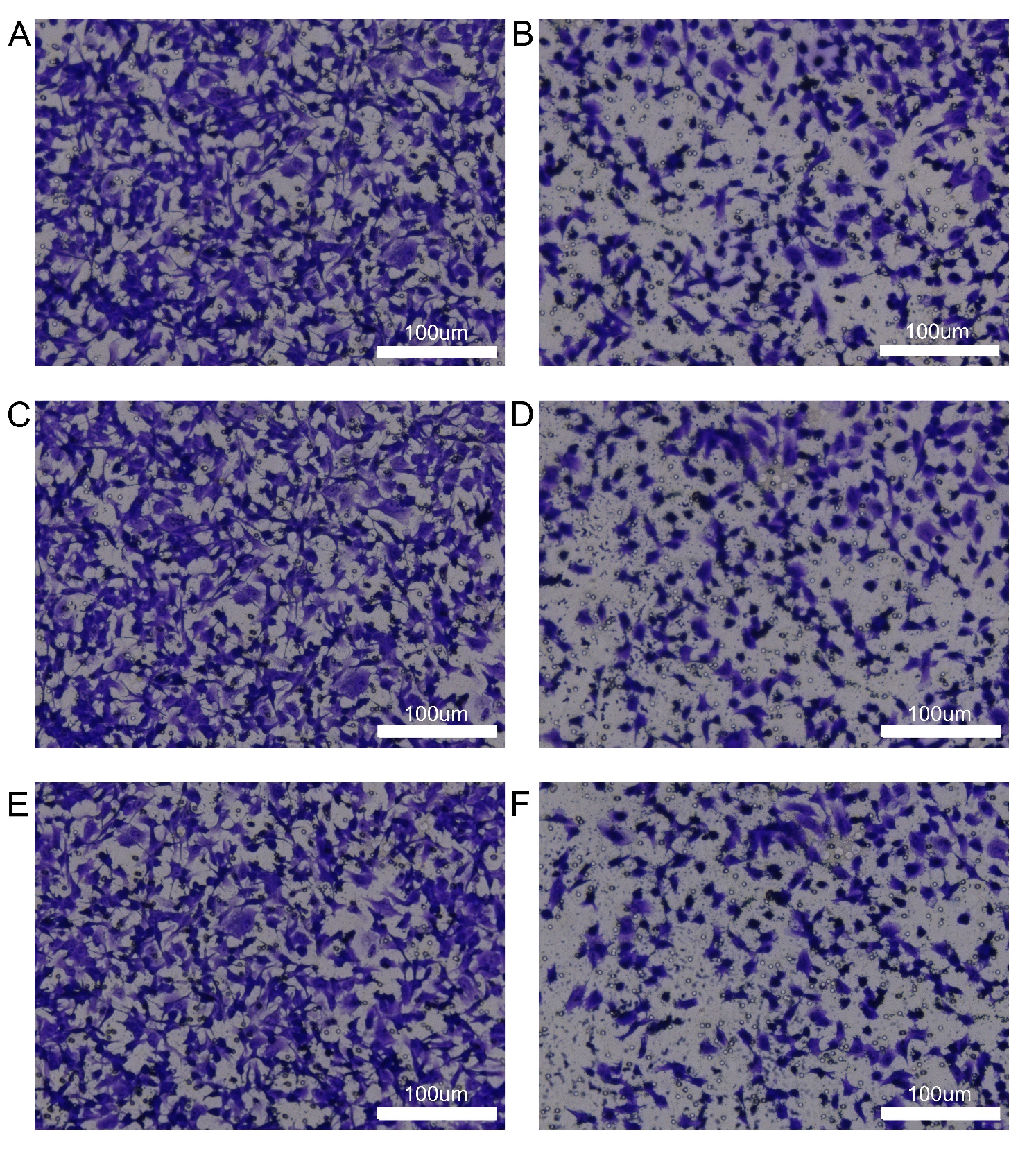
Supplementary Figure 6 | Transwell assay was used to examine the cell invasion ability of si-NC (A, C, E) and si-MGLL (B, D, F) UM cells. A-B, C-D, E-F are three independent experiments. Scale bar represents 100 μm.

Supplementary Figure 7 | Wound-healing assay was used to examine the cell invasion ability of si-NC and si-MGLL UM cells. A, B, and C are three independent experiments. Scale bar represents 200 μm.
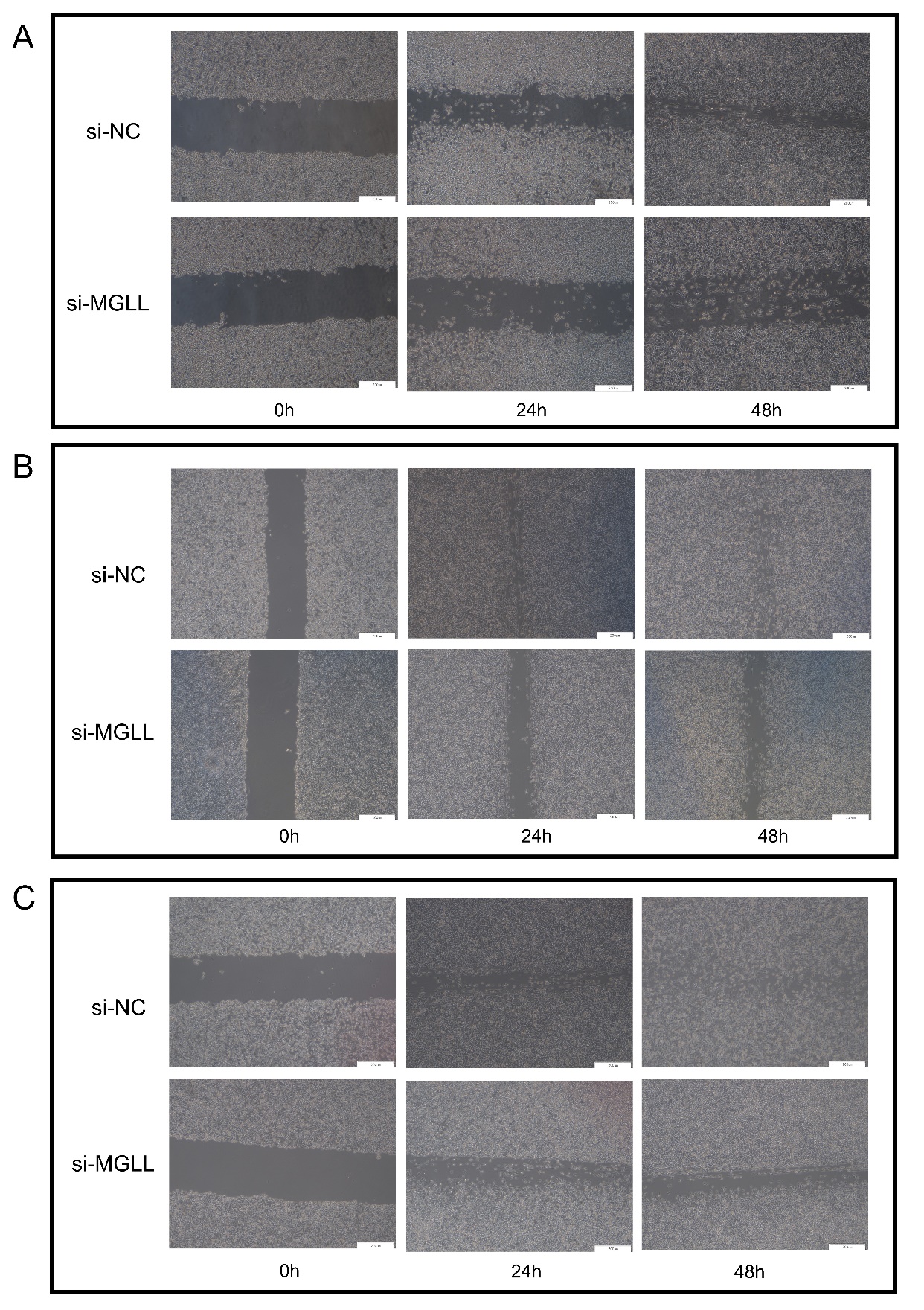


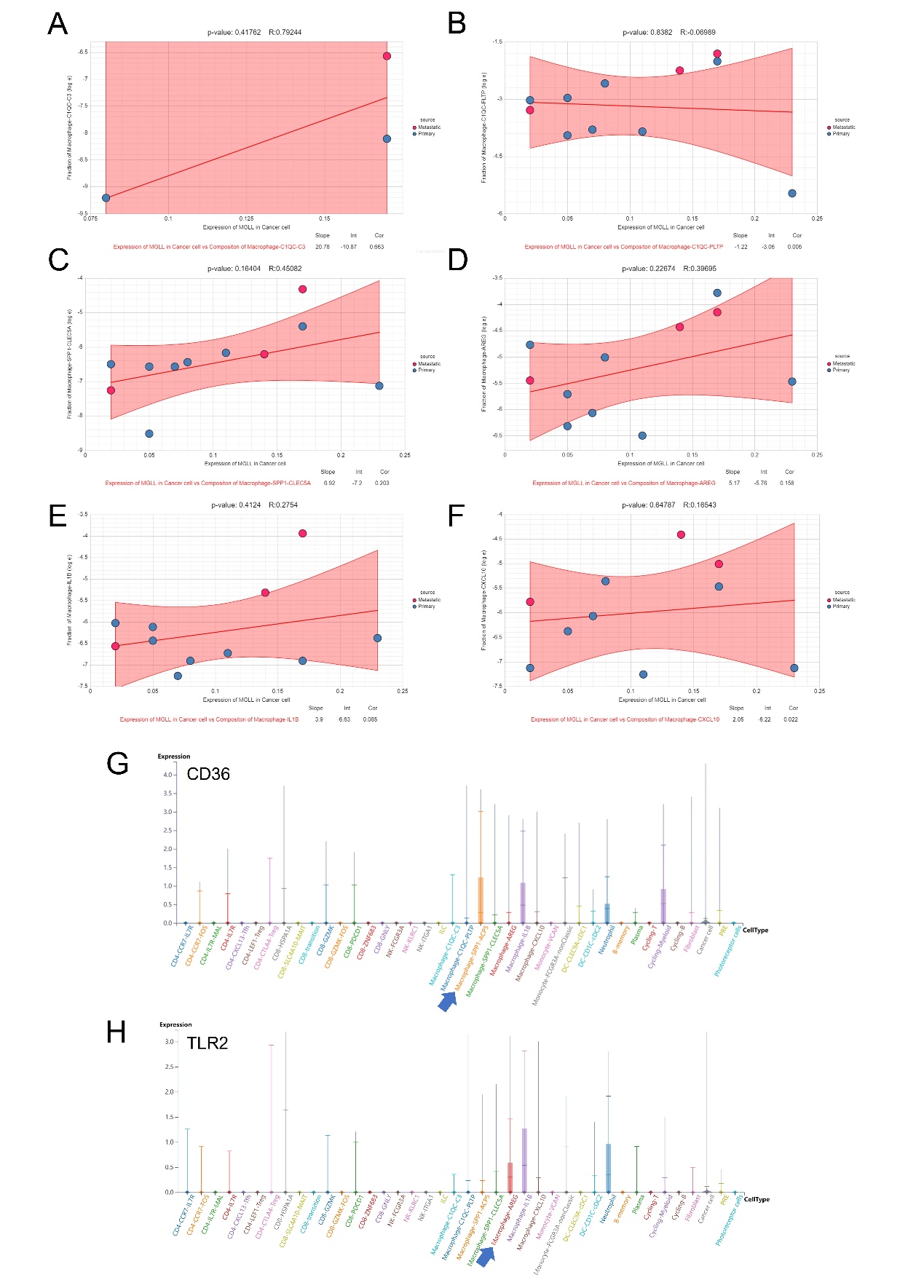
Supplementary Figure 8 | Effect of MGLL on infiltrating macrophages of TME. Correlation analysis between expression of MGLL in cancer cells and composition of infiltrating macrophages (C1QC-C3) (A), macrophages (C1QC-PLTP) (B), macrophages (SPP1-CLEC5A) (C), macrophages (AREG) (D), macrophages (IL1B) (E), and macrophages (CXCL10) (F). (G) CD36 expression in distinct clusters of cells. (H) TLR2 expression in distinct clusters of cells.

## Supplementary Tables

Supplementary Table 1 | The detailed lipid metabolism-related gene sets from the GSEA and KEGG database.

| **Database** | **Gene Set** | **Gene Count** |
| --- | --- | --- |
| GSEA | GOBP_PHOSPHOLIPID_METABOLIC_PROCESS | 389 |
|  | GOBP_REGULATION_OF_CHOLESTEROL_METABOLIC_PROCESS | 38 |
|  | HALLMARK_FATTY_ACID_METABOLISM | 158 |
|  | KEGG_FATTY_ACID_METABOLISM | 42 |
|  | KEGG_GLYCEROLIPID_METABOLISM | 49 |
|  | KEGG_GLYCEROPHOSPHOLIPID_METABOLISM | 77 |
|  | KEGG_SPHINGOLIPID_METABOLISM | 39 |
|  | REACTOME_FATTY_ACID_METABOLISM | 177 |
|  | REACTOME_GLYCOSPHINGOLIPID_METABOLISM | 45 |
|  | REACTOME_KETONE_BODY_METABOLISM | 10 |
|  | REACTOME_METABOLISM_OF_LIPIDS | 743 |
|  | REACTOME_PHOSPHOLIPID_METABOLISM | 211 |
|  | REACTOME_REGULATION_OF_LIPID_METABOLISM_LISM_BY_PPARALPHA | 120 |
|  | REACTOME_SPHINGOLIPID_METABOLISM | 90 |
|  | REACTOME_TRANSCRIPTIONAL_REGULATION_OF_W_OF_WHITE_ADIPOCYTE_DIFFERENTIATION | 84 |
|  | WP_CHOLESTEROL_METABOLISM_WITH_BLOCH_AND_KANDUTSCHRUSSELL_PATHWAYS | 48 |
|  | WP_LIPID_METABOLISM_PATHWAY | 29 |
| KEGG | hsa00061 Fatty acid biosynthesis | 18 |
|  | hsa00062 Fatty acid elongation | 27 |
|  | hsa00071 Fatty acid degradation | 43 |
|  | hsa00072 Synthesis and degradation of ketone bodies | 43 |
|  | hsa00100 Steroid biosynthesis | 20 |
|  | hsa00120 Primary bile acid biosynthesis | 17 |
|  | hsa00140 Steroid hormone biosynthesis | 61 |
|  | hsa00561 Glycerolipid metabolism | 61 |
|  | hsa00564 Glycerophospholipid metabolism | 98 |
|  | hsa00565 Ether lipid metabolism | 49 |
|  | hsa00590 Arachidonic acid metabolism | 61 |
|  | hsa00591 Linoleic acid metabolism | 29 |
|  | hsa00592 alpha-Linolenic acid metabolism | 25 |
|  | hsa00600 Sphingolipid metabolism | 53 |
|  | hsa01040 Biosynthesis of unsaturated fatty acids | 27 |
|  | hsa04979 Cholesterol metabolism | 50 |
| Sum |  | 3031(unique:1168) |

Supplementary Table 2 | Abbreviations for all cancer types.

| Abbreviation | Full name |
| --- | --- |
| ACC | Adrenocortical carcinoma |
| ALL | Acute Lymphoblastic Leukemia |
| BLCA | Bladder Urothelial Carcinoma |
| BRCA | Breast invasive carcinoma |
| CESC | Cervical squamous cell carcinoma and endocervical adenocarcinoma |
| CHOL | Cholangiocarcinoma |
| COAD | Colon adenocarcinoma |
| COADREAD | Colon adenocarcinoma/Rectum adenocarcinoma Esophageal carcinoma |
| DLBC | Lymphoid Neoplasm Diffuse Large B-cell Lymphoma |
| ESCA | Esophageal carcinoma |
| FPPP | FFPE Pilot Phase II |
| GBM | Glioblastoma multiforme |
| GBMLGG | Glioma |
| HNSC | Head and Neck squamous cell carcinoma |
| KICH | Kidney Chromophobe |
| KIPAN | Pan-kidney cohort (KICH+KIRC+KIRP) |
| KIRC | Kidney renal clear cell carcinoma |
| KIRP | Kidney renal papillary cell carcinoma |
| LAML | Acute Myeloid Leukemia |
| LGG | Brain Lower Grade Glioma |
| LIHC | Liver hepatocellular carcinoma |
| LUAD | Lung adenocarcinoma |
| LUSC | Lung squamous cell carcinoma |
| MESO | Mesothelioma |
| NB | Neuroblastoma |
| OS | Osteosarcoma |
| OV | Ovarian serous cystadenocarcinoma |
| PAAD | Pancreatic adenocarcinoma |
| PCPG | Pheochromocytoma and Paraganglioma |
| PRAD | Prostate adenocarcinoma |
| READ | Rectum adenocarcinoma |
| SARC | Sarcoma |
| SKCM | Skin Cutaneous Melanoma |
| STAD | Stomach adenocarcinoma |
| STES | Stomach and Esophageal carcinoma |
| TGCT | Testicular Germ Cell Tumors |
| THCA | Thyroid carcinoma |
| THYM | Thymoma |
| UCEC | Uterine Corpus Endometrial Carcinoma |
| UCS | Uterine Carcinosarcoma |
| UVM | Uveal Melanoma |
| WT | High-Risk Wilms Tumor |

Supplementary Table 3 | Primer sequences used for real-time quantitative PCR (q-PCR) analysis.

| **Gene** | **5' to 3'** | **Primers** |
| --- | --- | --- |
| MGLL | Forward Sequence | CAAGGCCCTCATCTTTGTGT |
| MGLL | Reverse Sequence | ACGTGGAAGTCAGACACTAC |
| ENPP2 | Forward Sequence | CTCACCCTGCAGATCATGA |
| ENPP2 | Reverse Sequence | CTCAGTTCTATCACATGTGAC |
| PLCD1 | Forward Sequence | CAGCGTCAGAAGCTACAGCA |
| PLCD1 | Reverse Sequence | TCTGTCTGGGAGTGGTCACA |
| SLC44A3 | Forward Sequence | GGCAATGTGTGTGGCAAGAG |
| SLC44A3 | Reverse Sequence | AGAGTCATGTCCTGCCCAGAGA |
| GAPDH | Forward Sequence | GGAGCGAGATCCCTCCAAAAT |
| GAPDH | Reverse Sequence | GGCTGTTGTCATACTTCTCATGG |
| CD36 | Forward Sequence | CTTTGGCTTAATGAGACTGGGAC |
| CD36 | Reverse Sequence | GCAACAAACATCACCACACCA |
| TLR2 | Forward Sequence | ATCCTCCAATCAGGCTTCTCT |
| TLR2 | Reverse Sequence | GGACAGGTCAAGGCTTTTTACA |

Supplementary Table 4 | Univariate Cox regression results of 47 lipid metabolism-related genes and survival.

| **Gene** | **HR** | **HR.95L** | **HR.95H** | **P value** |
| --- | --- | --- | --- | --- |
| ABHD3 | 2.3447737 | 1.429189031 | 3.846911489 | 0.000384574 |
| ACSF2 | 0.374046448 | 0.221309634 | 0.632194553 | 6.79786E-05 |
| ACSL1 | 1.873686292 | 1.236619189 | 2.838950221 | 0.00241788 |
| APOE | 0.658151562 | 0.456263704 | 0.949370888 | 0.024428243 |
| BCHE | 0.363227956 | 0.187094993 | 0.705174123 | 0.000812947 |
| CEBPD | 2.26328452 | 1.425983183 | 3.592228071 | 0.000257659 |
| DECR1 | 3.425630666 | 1.935233597 | 6.063839258 | 4.1792E-06 |
| DHRS7B | 4.620295169 | 2.167694549 | 9.847848471 | 1.25798E-05 |
| EFR3A | 1.781309052 | 1.186992897 | 2.673193705 | 0.004138391 |
| ENPP2 | 0.406330979 | 0.271636142 | 0.607816263 | 1.88747E-07 |
| EPHX2 | 0.348229258 | 0.192562698 | 0.629735754 | 0.000264441 |
| FABP3 | 2.281173597 | 1.469222252 | 3.541841933 | 6.1956E-05 |
| FABP5 | 2.67353739 | 1.724188773 | 4.145603015 | 1.96055E-06 |
| FADS1 | 4.601125566 | 2.228984088 | 9.497760255 | 9.07086E-06 |
| FDFT1 | 0.43744748 | 0.279716796 | 0.684121584 | 0.000166669 |
| GGT1 | 2.16234964 | 1.495484536 | 3.126582626 | 7.28348E-06 |
| HACD1 | 4.273693684 | 2.044098349 | 8.935214747 | 2.15274E-05 |
| HEXB | 4.689149551 | 1.963593145 | 11.19790195 | 0.000177638 |
| HPGD | 0.469285269 | 0.269142798 | 0.818259543 | 0.002706807 |
| HSD17B8 | 0.379501715 | 0.224076652 | 0.642733416 | 0.000122719 |
| HTR2B | 1.453577216 | 1.224078472 | 1.726103981 | 1.02495E-06 |
| IMPA1 | 2.115178641 | 1.434983112 | 3.117793266 | 6.2482E-05 |
| INPP4B | 1.864927959 | 1.152846086 | 3.016843563 | 0.010350851 |
| KLF4 | 1.664199328 | 1.122298497 | 2.467756492 | 0.009421785 |
| LGALS1 | 2.763341637 | 1.646532703 | 4.637658875 | 4.29836E-05 |
| LYPLA1 | 1.729923729 | 1.172409832 | 2.552551186 | 0.004470847 |
| ME1 | 1.806568577 | 1.259836699 | 2.590565925 | 0.000649927 |
| MGLL | 1.862811105 | 1.443259818 | 2.404324689 | 3.72468E-07 |
| NBN | 1.862150543 | 1.232352768 | 2.813808459 | 0.002289604 |
| OXCT1 | 1.998497682 | 1.203155317 | 3.319598832 | 0.006228406 |
| PIK3C2A | 1.739799838 | 1.233762081 | 2.453393181 | 0.001116812 |
| PIP4P2 | 1.931143559 | 1.174022137 | 3.176529067 | 0.008607932 |
| PLA1A | 0.728157886 | 0.549828076 | 0.964326724 | 0.023956954 |
| PLAAT1 | 0.19946867 | 0.07691273 | 0.517310339 | 0.000426275 |
| PLAAT4 | 1.313615099 | 1.070839494 | 1.611431627 | 0.007280883 |
| PLCD1 | 0.146407396 | 0.06324262 | 0.338934812 | 1.52315E-07 |
| PLIN2 | 0.372587934 | 0.230460041 | 0.602368062 | 2.4113E-05 |
| PPARG | 0.52151403 | 0.332253343 | 0.818582833 | 0.003029472 |
| PRKCD | 0.265343472 | 0.139333031 | 0.505315631 | 1.40009E-05 |
| SLC44A3 | 0.331299512 | 0.209866447 | 0.522996258 | 3.34225E-08 |
| SNCA | 0.730231962 | 0.537741573 | 0.991626361 | 0.040612591 |
| SOAT1 | 2.241922824 | 1.388734655 | 3.619278838 | 0.000467114 |
| SPHK1 | 2.03787598 | 1.353082782 | 3.069242003 | 0.000452946 |
| SQLE | 2.000467246 | 1.425076543 | 2.808178425 | 1.65859E-05 |
| SRD5A3 | 2.620827217 | 1.58163711 | 4.342801049 | 7.70083E-05 |
| TGS1 | 1.888727333 | 1.235029722 | 2.888425173 | 0.002547896 |
| TNFRSF21 | 2.373024349 | 1.479703092 | 3.805658441 | 0.000140879 |

Supplementary Table 5 | The enrichment analysis was carried out by Metascape.

| **MCODE** | **GO** | **Description** | **Log10(P)** |
| --- | --- | --- | --- |
| MCODE_1 | WP545 | Complement activation | -14.9 |
| MCODE_1 | R-HSA-166663 | Initial triggering of complement | -14.7 |
| MCODE_1 | R-HSA-977606 | Regulation of Complement cascade | -14.5 |
| MCODE_2 | R-HSA-913531 | Interferon Signaling | -37.5 |
| MCODE_2 | hsa05330 | Allograft rejection | -31.4 |
| MCODE_2 | GO:0048002 | antigen processing and presentation of peptide antigen | -30.8 |
| MCODE_3 | R-HSA-72766 | Translation | -18.4 |
| MCODE_3 | hsa03010 | Ribosome | -18.3 |
| MCODE_3 | GO:0006412 | translation | -17.8 |
| MCODE_4 | R-HSA-8873719 | RAB geranylgeranylation | -8 |
| MCODE_4 | hsa04260 | Cardiac muscle contraction | -5.3 |
| MCODE_4 | WP111 | Electron transport chain: OXPHOS system in mitochondria | -5 |
| MCODE_5 | GO:0002761 | regulation of myeloid leukocyte differentiation | -7.6 |
| MCODE_5 | R-HSA-9031628 | NGF-stimulated transcription | -6.8 |
| MCODE_5 | R-HSA-9006934 | Signaling by Receptor Tyrosine Kinases | -6.7 |
| MCODE_6 | hsa00480 | Glutathione metabolism | -12.3 |
| MCODE_6 | R-HSA-156590 | Glutathione conjugation | -7.3 |
| MCODE_6 | GO:0006749 | glutathione metabolic process | -6.5 |
| MCODE_7 | hsa05200 | Pathways in cancer | -4.3 |
| MCODE_7 | GO:0071417 | cellular response to organonitrogen compound | -4.2 |
| MCODE_7 | GO:1901699 | cellular response to nitrogen compound | -4.1 |
| MCODE_8 | R-HSA-9660821 | ADORA2B mediated anti-inflammatory cytokines production | -9.4 |
| MCODE_8 | R-HSA-418555 | G alpha (s) signalling events | -9.3 |
| MCODE_8 | R-HSA-9664433 | Leishmania parasite growth and survival | -9 |
| MCODE_9 | R-HSA-936837 | Ion transport by P-type ATPases | -10.9 |
| MCODE_9 | R-HSA-983712 | Ion channel transport | -8.9 |
| MCODE_9 | R-HSA-5578775 | Ion homeostasis | -7.6 |
| MCODE_10 | R-HSA-5668541 | TNFR2 non-canonical NF-kB pathway | -7.4 |
| MCODE_10 | R-HSA-1280215 | Cytokine Signaling in Immune system | -4.9 |
| MCODE_11 | R-HSA-416476 | G alpha (q) signalling events | -6.4 |
| MCODE_11 | hsa04020 | Calcium signaling pathway | -6.3 |
| MCODE_11 | WP455 | GPCRs, class A rhodopsin-like | -6.2 |
| MCODE_12 | R-HSA-6798695 | Neutrophil degranulation | -5.4 |
| MCODE_13 | hsa04510 | Focal adhesion | -6.5 |
| MCODE_13 | WP306 | Focal adhesion | -6.5 |
| MCODE_13 | hsa04015 | Rap1 signaling pathway | -6.5 |
| MCODE_14 | hsa00230 | Purine metabolism | -7.1 |

Supplementary Table 6 | MGLL expression and tumor purity correlation in pan-cancer.

| **Cancer** | **Full name** | **Sample size** | **R value** | **P value** |
| --- | --- | --- | --- | --- |
| PCPG | Pheochromocytoma and Paraganglioma | 160 | -0.588314227 | 2.83144960607441e-16 |
| UVM | Uveal Melanoma | 79 | -0.335804449 | 0.00248224836672502 |
| DLBC | Lymphoid Neoplasm Diffuse Large B-cell Lymphoma | 46 | -0.330012742 | 0.0251027264883529 |
| KIPAN | Pan-kidney cohort (KICH+KIRC+KIRP) | 844 | -0.309538833 | 3.37426136394281e-20 |
| LUSC | Lung squamous cell carcinoma | 490 | 0.305509961 | 4.7988423214718e-12 |
| HNSC | Head and Neck squamous cell carcinoma | 505 | -0.304051078 | 2.91558337164536e |
| BLCA | Bladder Urothelial Carcinoma | 397 | -0.295627742 | 1.89519381976471e-9 |
| LAML | Acute Myeloid Leukemia | 111 | -0.278372536 | 0.00309295845663578 |
| PRAD | Prostate adenocarcinoma | 470 | -0.222881144 | 0.00000105759841805965 |
| LGG | Brain Lower Grade Glioma | 503 | -0.220417524 | 5.95458582231861e-7 |
| GBMLGG | Glioma | 646 | -0.183474525 | 0.00000267705366246237 |
| KIRP | Kidney renal papillary cell carcinoma | 283 | -0.168266867 | 0.00453368557601075 |
| LIHC | Liver hepatocellular carcinoma | 356 | -0.166487693 | 0.00162009698279763 |
| TGCT | Testicular Germ Cell Tumors | 147 | -0.165088899 | 0.045690426367015 |
| UCEC | Uterine Corpus Endometrial Carcinoma | 178 | -0.163632398 | 0.0290741453954318 |
| BRCA | Breast invasive carcinoma | 1043 | -0.149499257 | 0.00000123683480303413 |
| KIRC | Kidney renal clear cell carcinoma | 495 | -0.126859993 | 0.00470230547469252 |
| STES | Stomach and Esophageal carcinoma | 560 | -0.124146757 | 0.00325389225675117 |
| OV | Ovarian serous cystadenocarcinoma | 407 | -0.121461239 | 0.0142089841582894 |
| THCA | Thyroid carcinoma | 462 | -0.111713181 | 0.0162966798829064 |
| STAD | Stomach adenocarcinoma | 402 | -0.111112629 | 0.0258960907043343 |
| THYM | Thymoma | 102 | 0.214407576 | 0.0304678084633976 |
